# Mapping the perturbome network of cellular perturbations

**DOI:** 10.1038/s41467-019-13058-9

**Published:** 2019-11-13

**Authors:** Michael Caldera, Felix Müller, Isabel Kaltenbrunner, Marco P. Licciardello, Charles-Hugues Lardeau, Stefan Kubicek, Jörg Menche

**Affiliations:** 1 CeMM Research Center for Molecular Medicine of the Austrian Academy of Sciences, Lazarettgasse 14, AKH BT 25.3, A-1090 Vienna, Austria; 2 Present Address: Cancer Research UK Cancer Therapeutics Unit, The Institute of Cancer Research, London, UK; 3 Present Address: Hit Discovery, Discovery Sciences, R&D, AstraZeneca, Alderley Park, Macclesfield, UK

**Keywords:** Networks and systems biology, Network topology, Modularity, Regulatory networks

## Abstract

Drug combinations provide effective treatments for diverse diseases, but also represent a major cause of adverse reactions. Currently there is no systematic understanding of how the complex cellular perturbations induced by different drugs influence each other. Here, we introduce a mathematical framework for classifying any interaction between perturbations with high-dimensional effects into 12 interaction types. We apply our framework to a large-scale imaging screen of cell morphology changes induced by diverse drugs and their combination, resulting in a perturbome network of 242 drugs and 1832 interactions. Our analysis of the chemical and biological features of the drugs reveals distinct molecular fingerprints for each interaction type. We find a direct link between drug similarities on the cell morphology level and the distance of their respective protein targets within the cellular interactome of molecular interactions. The interactome distance is also predictive for different types of drug interactions.

## Abstract (web summary)

Our understanding of the mechanisms of drug interactions remains limited. Here the authors introduce a framework to study how complex cellular perturbations induced by different drugs affect each other in morphological feature space.

## Introduction

Biological function relies on the careful orchestration of numerous and diverse cellular components and their interactions. Disease states, but also therapeutic interventions, can be viewed as perturbations of this intricate system, either driving it away from homeostasis, or aiming to restore it, respectively. Understanding the combined effect of independent perturbations lies at the core of many fundamental, as well as practical challenges in current biology and medicine. Combination therapies, for example, provide promising new treatment strategies for diseases ranging from cancer to bacterial or viral infections^1,2^. At the same time, interactions between drugs and/or comorbidities may also induce unexpected side effects. Adverse reactions are a primary cause for the failure of clinical trials^3^ and represent a major challenge in drug development and repurposing^4–6^. Especially for elderly patients, for whom a combination of several disease conditions and simultaneous drug treatments is not uncommon, adverse effects can be a severe threat^7^. Both for avoiding such detrimental effects, and for rationally designing beneficial combinations of therapeutic and/or disease associated perturbations, we need to first understand how different perturbations interact with each other.

Molecular networks provide a unifying platform to systematically investigate the combined effects of perturbations of biological systems. In recent years, an increasingly detailed network diagram of the complex machinery of interacting molecules that constitutes the basis of (patho-) physiological states has become available^8,9^. Network-based analyses revealed an intimate relationship between structural properties of this *interactome* network of protein–protein interactions (PPIs), its functional organization, and consequences of its perturbation. The most essential cellular components, as well as genes associated with severe diseases, tend to be located at the center of the interactome^10,11^. Genes that are associated with the same disease aggregate in specific network neighborhoods, or “disease modules”^9,12,13^. The closeness of a drug’s target(s) to a disease module is related to its efficacy, but also to side effects that may occur^14,15^. This again highlights our lack of a systematic understanding of how independent perturbations influence each other.

Here, we set out to identify key principles of how different perturbations are integrated through the molecular network. First, we want to understand the directionality of this process, i.e. whether one perturbation increases or decreases the effect of another, for example, a drug that may alleviate or worsen a certain condition. Secondly, we want to understand the conditions under which emergent behavior occurs, i.e. when the combination of perturbations promotes entirely new outcomes, such as unexpected side effects. Lastly, we aim to connect either phenomena observed on a phenotypic level to underlying molecular-level determinants.

We address these questions using a combined theoretical and experimental approach: we develop a rigorous mathematical framework for defining and quantifying all possible interactions that may occur between perturbations that cause complex phenotypes. This framework is then applied to a large-scale imaging screen of a well-controlled cell line model system. High-content imaging is a powerful tool for profiling cellular perturbations in a detailed and unbiased fashion^16,17^. In some cases, morphological changes can be linked directly to specific molecular mechanisms, for example, for drugs that affect the cytoskeleton. In many other cases, however, the relation to cellular organization is less direct and remains to be understood^18–21^. We quantify the morphological changes induced by 267 individual drug compounds and all 35,611 pairwise combinations. We find that distinct cell morphologies can be associated with chemical perturbations of distinct neighborhoods within the molecular network and that the overlap between these neighborhoods is predictive for interactions between the respective perturbations.

## Results

### The interactome patterns of chemical perturbations

**Fig. 1:**
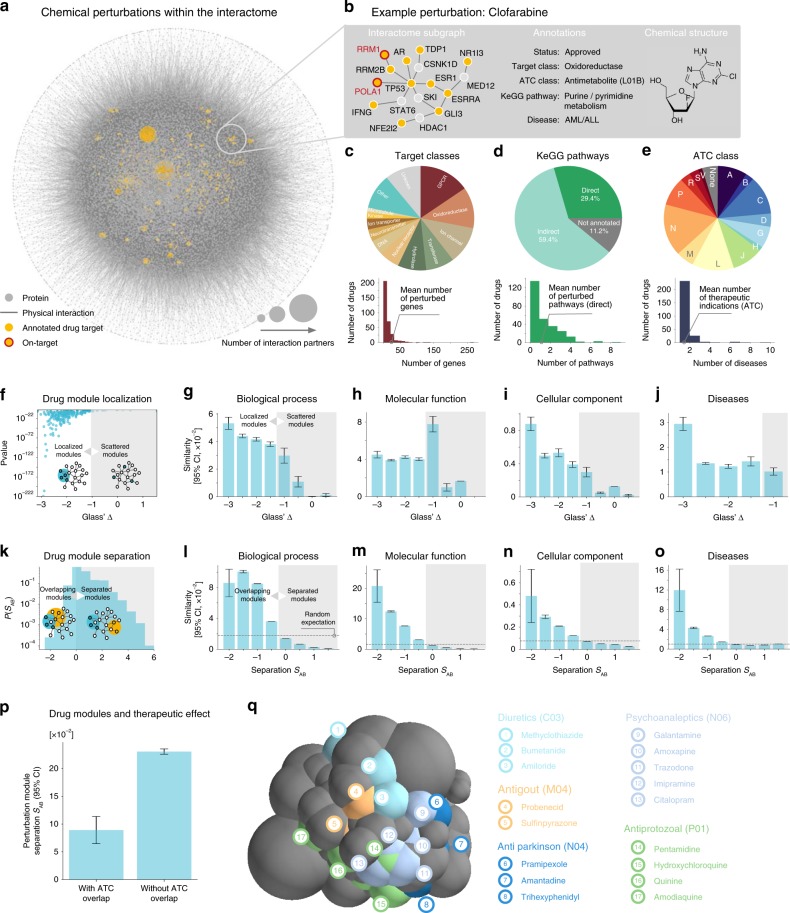
The perturbation landscape of chemical compounds in the human interactome. **a** Interactome consisting of 16,376 proteins and 309,355 physical interactions, with 1096 unique targets of the 267 drugs used in this study highlighted in yellow. **b** Exemplary subgraph for one drug and summary of collected annotations. **c**–**e** Drug library overview: covered classes of protein targets (**c**), affected pathways (**d**), and therapeutic classes (**e**). **f** Drug targets form localized modules in the interactome (Glass′ ∆ < −1 indicates strong localization). **g**–**j** Degree of localization correlates with similarity of biological processes (**g**), molecular function (**h**), cellular component (**i**), and known disease associations (**j**) of target proteins. **k** Distribution of interactome overlaps across all drug pairs (***S***_*AB*_<0 indicates overlapping modules, ***S***_*AB*_<0 separate ones). **l**–**o** Interactome overlap is associated with drug similarity in terms of biological processes (**l**), molecular function (**m**), cellular component (**n**), and known disease associations (**o**). **p** Drugs belonging to the same therapeutic class are characterized by overlapping interactome modules. The bars in **g**–**j**, **l**–**p** indicate the mean over all measurements, error bars show the 95% confidence interval. **q** Representation of interactome-based drug relationships in a 3D perturbation space. Each sphere represents a compound, the diameter is proportional to its interactome localization, the overlap between two spheres is proportional to their module overlap *S*_*AB*_. Five broad therapeutic disease classes are indicated in color. Modules of drugs that are used to treat similar diseases are co-located. We also observe overlaps between modules of drugs that are used to treat a particular disease and modules of drugs in which the respective disease may occur as a side effect

We started by compiling a comprehensive interactome consisting of 309,355 physical interactions between 16,376 proteins (Fig. 1a, Supplementary Fig. 1, Supplementary Data 1 and Methods section). To perturb the interactome, we used a library of 267 chemical compounds, of which 256 are approved for clinical use (Fig. 1b, Supplementary Data 2 and 3 and Methods section). The library was designed to represent a wide range of mechanisms of action (MOAs), structural diversity, and targeted biological processes (Fig. 1c–e, Supplementary Fig. 2, Supplementary Data 4 and Supplementary Methods for a characterization of the CLOUD library). The mean number of protein targets per compound is 13.64, but there are also several compounds with over 100 targets (Supplementary Data 5). Taken together, 32% of all molecular pathways contained in the KeGG database are annotated as directly related to the MOA of a particular compound; 89% contain at least one targeted protein. The targets are characterized by a broad distribution of the number of interactors, or degree *k*, similar to the one of the full interactome, albeit with a significant tendency towards more highly connected proteins (〈*k*_all_〉 = 37.7 versus 〈*k*_targets_〉 = 74.4; Supplementary Fig. 3). While this is likely the consequence of being more extensively studied, in part it may also reflect a higher propensity of more highly connected proteins to yield a response upon perturbation, i.e., a therapeutic effect.

The connectivity patterns between the targets of a given compound show striking similarities to patterns observed among disease associated genes. First, they tend to aggregate in specific interactome neighborhoods, or “perturbation modules”: 64% of the compounds target proteins that form connected subgraphs within the interactome that are significantly larger than expected by chance; 92% of all compounds are characterized by significantly shorter interactome distances *d*_s_ between their associated targets. We quantified the degree of interactome localization using Glass′ ∆, which compares the observed interactome distances between targets to those obtained from randomly sampled genes (Fig. 1f, Supplementary Fig. 4 and Methods section). Second, there is a strong correlation between the degree of interactome localization and the biological similarity of the respective proteins: the average functional similarity in terms of Gene Ontology (GO) annotations is up to 32-fold higher for strongly localized perturbation modules (Glass′ ∆ ≤ −3) than for perturbation modules whose targets are randomly scattered over the interactome (Glass′ ∆ ≥ 0) (Fig. 1g–i, Supplementary Fig. 5A–C). The same trend was observed when considering known disease associations of the target proteins: highly localized modules are associated with cohesive disease phenotypes, whereas more widespread modules are related to more heterogeneous groups of diseases (Fig. 1j). Third, the interactome-based overlap between two perturbation modules is indicative of shared cellular processes and disease associations (Fig. 1k–o, Supplementary Fig. 5D). Finally, we recapitulate previous findings that drugs targeting close by interactome neighborhoods tend to share therapeutic usage and side effects^15,22^ (Fig. 1p, Supplementary Fig. 5E, F).

The interactome characteristics of perturbation modules and their relationships can be summarized in a three-dimensional perturbation space (Fig. 1q). Each perturbation module is represented by a sphere in this space, such that distances and overlaps between spheres approximate the respective quantities measured on the interactome. Compounds used to treat the same disease class tend to be close within the perturbation space, as shown in Fig. 1q for anti-Parkinson drugs (ATC, Anatomical Therapeutic Chemical Classification System, class N04) or psychoanaleptics (N06). Interestingly, the perturbation space also revealed a closeness between treatments and related side effects. For example, anti-protozoal drugs, which have been associated with psychoactive side effects, overlapped with analeptics that stimulate the central nervous system^23^. Similarly, the proximity between anti-gout medications (M05) and diuretics reflects a clinically observed relationship, as the side effects of diuretics include hypercalcemia and hyperuricemia, which in turn are closely related to gout^24^. These examples highlight again the importance of a deeper understanding of the precise interplay between interactome perturbations. To disentangle whether two proximal perturbations represent either a pathobiological perturbation and a potential treatment, or a treatment and a potential side effect, we must understand the directionality of their interplay. In other words, whether the effects of one perturbation are increased or decreased by a second perturbation, or whether their combination leads to yet another, unexpected outcome.

### Quantifying high-dimensional perturbation interactions

**Fig. 2:**
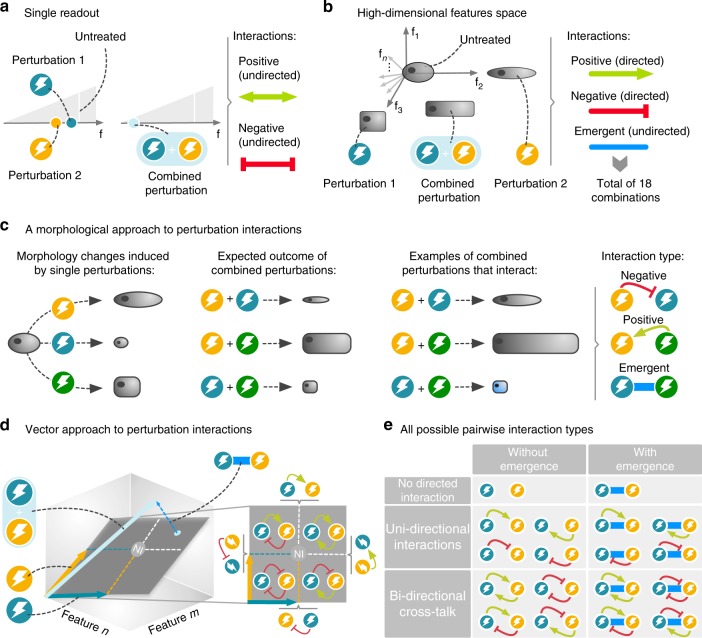
Mathematical framework for a complete description of pairwise perturbation interactions. **a** Single readout measurements (number of features *f* = 1, e.g. cell viability) can only distinguish three interaction types between perturbations: positive, negative (e.g., synergy/antagonism), or non-interaction. **b** High-dimensional readouts (*f* *>* 2, e.g. cell morphology), enable the identification of two directed interaction types (positive and negative), describing whether the effect of a perturbation was increased or decreased, as well as one undirected type, describing emergent features that are not present in any of the individual perturbations. **c** Each perturbation (i.e., drug treatment) is associated with a specific cell phenotype. When two perturbations are combined, we identify the superposition of their individual effects as non-interaction, and any deviation as interaction, respectively. **d** Perturbations can be represented as vectors in the high-dimensional feature space, pointing from the unperturbed to the perturbed state. Two perturbations span a 2D plane *S*, which also contains the expected non-interaction (NI) phenotype. The NI point divides *S* into four quadrants that can be identified with different interaction types. Any measured phenotype of two combined perturbations can be decomposed into two components within *S*, which determine the directed interactions, and a third component perpendicular to *S*, indicating the emergence of entirely new features. **e** Summary of all 18 possible interaction types that may occur between two perturbations

To address these questions systematically, we first had to devise a mathematical framework that can fully capture the diversity of mutual interactions that may arise between perturbations with complex responses. Existing methodologies focus on a single readout only, most notably cellular toxicity^25,26^. While cell death (or survival, respectively) represents the key outcome for many applications, such as cancer treatment or antibiotics, complex biological systems like cells or whole organisms clearly have a much richer repertoire of responses to perturbations than being dead or not. Considering only a single readout fundamentally limits the modes of interactions that can be observed. Depending on whether a response is more, less or equally pronounced as expected, one can only define *synergy*, *antagonism*, or *non-interaction*, respectively (Fig. 2a). Higher-dimensional readouts, in contrast, allow for a much more detailed characterization of perturbations and their interactions^27,28^. As we show below, high-dimensional readouts can be used to extract not only the type of an interaction but also its direction (Fig. 2b). In the following, we will focus on readouts describing detailed morphological profiles as extracted from our experimental system. However, our framework can be applied to arbitrary stimuli, for example, gene knockouts, as well as other high-dimensional readouts, such as gene expression profiles.

We start by characterizing a given cell shape by a set of morphological features (Fig. 2c), which in turn represents a point within the high-dimensional morphological space of all possible shapes. A perturbation that changes the shape can then be identified with a unique vector $\vec A$ that points from the unperturbed (DMSO treated) to the perturbed (drug treated) state (Fig. 2d and Methods section). Even for arbitrary high dimensionality of the full state space, two perturbations $\vec A$ and $\vec B$ can always be embedded into a two-dimensional plane *S*. By definition, perturbations that do not interfere with each other are expected to result in a simple superposition ${\overrightarrow {AB}}_{\mathrm{ind}} = \vec A + \vec B$ of the individual effects. Any deviation between this expectation and an experimentally observed cell state of two combined perturbations ${\overrightarrow{AB}}_{\mathrm{obs}}$ can be identified with an interaction that occurred between them. Mathematically, any combination vector can be uniquely decomposed into three components (Supplementary Fig. 6): two components lie within *S* and represent the contributions of the two individual perturbations, potentially stretched or shortened, the third component points outside of *S*, representing an entirely new, emergent phenotype. This decomposition allows us to quantify precisely how the effect of each individual perturbation has been increased or decreased by the presence of the second perturbation, as well as whether their combination led to an emergent effect that cannot be attributed to either individual perturbation, but is purely a result of their combination. Taken together, these components offer a complete description of any potential outcome that may arise from combining two perturbations in terms of exactly 18 possible classes (Fig. 2e). The classes can be categorized into (i) two undirected patterns with either no interaction at all or only emergent interaction, (ii) eight uni-directional patterns where only one of two perturbations is modulated and (iii) eight bi-directional patterns where both perturbations affect each other.

### A morphology-based perturbation interaction screen

**Fig. 3:**
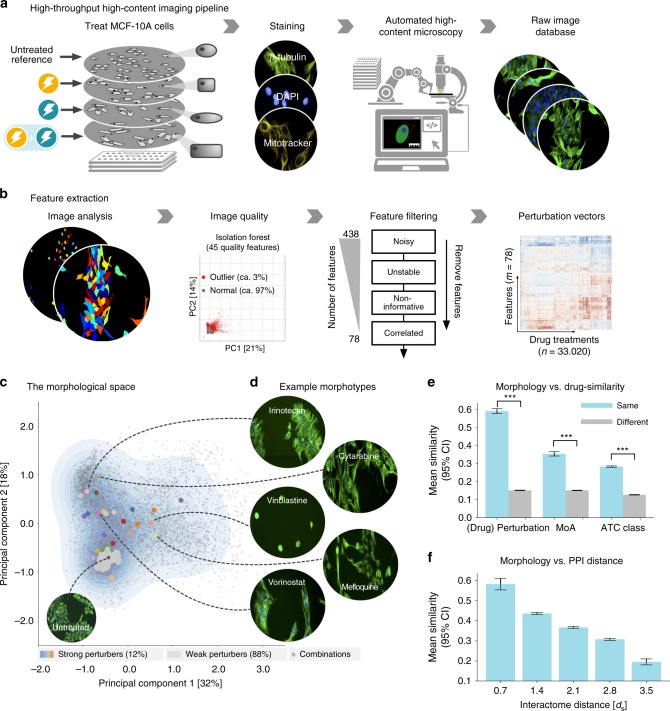
Imaging screen for identifying cell morphology changes induced by chemical perturbations and their combinations. **a** Overview of the imaging pipeline. We treated an epithelial MCF-10A cell line with 267 chemical compounds and all 35,511 pairwise combinations. Cells were plated in a 384-well plate, stained for nucleus, cytoskeleton, and mitochondria and imaged using automated high-throughput microscopy at 20× resolution. **b** Extraction of morphological features: images with technical artifacts are identified and removed using a machine learning approach. Cells are segmented and features are extracted using the software CellProfiler. We remove all features that are too noisy, not robust across replicates, show too little variation or are redundant, resulting in a 78-dimensional feature vector for each treatment. **c** The 78-dimensional morphological space projected onto its first two principal components. Each dot represents the cell morphology of a specific treatment (DMSO control, single drug, or drug combination). **d** Example images of drugs resulting in a strong morphological phenotype. **e** Evaluation of the morphological similarity for replicates of the same drug, drugs with the same mechanism of action (MOA) and drugs of the same therapeutic ATC class (*** denotes *P* value < 0.001, Mann–Whitney *U* test). **f** Morphological similarity versus interactome distance of the respective drug targets. The closer the targets of two drugs are on the interactome, the more similar are the morphological changes they induce in our cell line. Bars in **e**, **f** indicate the mean over all measurements; error bars show the 95% confidence interval

We used the human epithelial cell line MCF10-A and an experimental setup established previously for quantifying morphological changes upon drug treatment (Fig. 3a, Supplementary Data 6 and Methods section). We collected fluorescent microscopy images of cells treated with every individual compound in our library, as well as all pairwise combinations. Using the CellProfiler software^29^, we extracted a total of 438 morphological features (Supplementary Data 7 and 8) for each treatment, representing both intuitive geometric attributes (e.g., size, diameter), as well as more abstract mathematical descriptions of cell shape (e.g., Zernike polynomials). After filtering for the most robust, informative and non-redundant features, we obtained a final set of 78 morphological features, which thus define a 78-dimensional morphological space (Supplementary Data 9). The position of a specific treatment within the morphological space is determined by averaging the values of each feature across all cells of the respective treatment (Fig. 3b and Methods section).

After quality control, 28 out of 242 compounds showed a strong phenotype compared to untreated controls, a ratio consistent with previous reports^21^ (Supplementary Fig. 7, Supplementary Data 10 and Methods section). Note that for any individual morphological feature, even strong perturbations rarely result in a complete separation between the values observed in the treated and untreated cell populations, respectively. We therefore determine the strength of a given perturbation by the significance of the shift of its feature distributions relative to the respective distributions in the untreated cell population. We further evaluated the morphological heterogeneity, finding that in general, treated and untreated cells are characterized by similar levels of morphological variability. Neither showed evidence for the existence of significant subpopulations of cells with clearly distinct phenotype. We conclude that the observed morphological heterogeneity is largely driven by natural variation at the cellular level, and not by population level factors, for example that a particular drug only acted on a subset of cells (see Supplementary Methods and Supplementary Fig. 8 for a detailed analysis of cell population heterogeneity).

A principal component analysis (PCA) on the 78-dimensional morphological space shows the rich repertoire of cell morphologies (Fig. 3c, d, see also Supplementary Methods and Supplementary Fig. 9 for a comparison between visual similarity and closeness within the morphological space). For example, drugs targeting microtubules (e.g., vinblastine) resulted in small round cells, while antimetabolite drugs (e.g., cytarabine) often resulted in larger and branched cells. While some of the observed phenotypes can be linked directly to the molecular action of the respective drug, such as the tubulin inhibitors that disrupt the cytoskeleton, thus resulting in small, round phenotypes, others likely reflect more complex and integrated effects in several cellular components. Drug combinations further expand and fill this morphological space (Fig. 3c). The observed morphologies are highly robust, showing an average cosine similarity *S*_cos_ = 0.59 between replicates compared to *S*_cos_ = 0.15 between different drugs (Fig. 3e, Supplementary Fig. 10B). Our experiments further confirmed previous studies^19,30^ showing that morphological similarities can be related to similar MOAs (*S*_cos_ = 0.35 versus *S*_cos_ = 0.15) and, to a lesser extent, even to a common therapeutic class (*S*_cos_ = 0.28 versus *S*_cos_ = 0.13, Fig. 3e, Supplementary Fig. 10C, D).

Importantly, our analyses revealed a novel, direct link between drug similarities on the cell morphology level, and the interactome-based distance of their respective targets: as the network distance between targets increases, the morphological similarity gradually decreases from *S*_cos_ = 0.40 to *S*_cos_ = 0.07, i.e. by a total factor of 5.7, indicating that perturbations of specific interactome areas are related to specific morphologies (Fig. 3f, Supplementary Fig. 10E, F).

### The perturbome interaction network

**Fig. 4:**
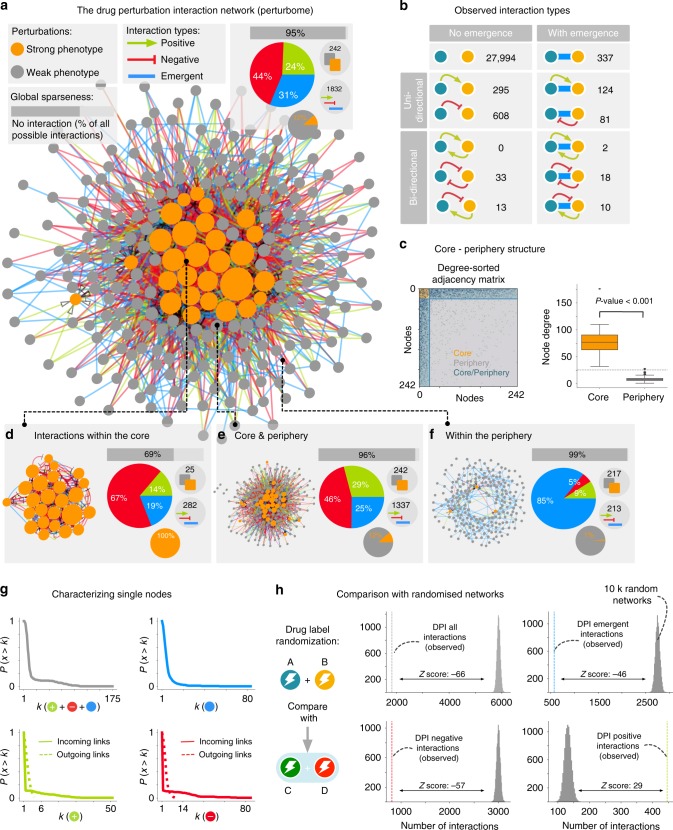
The perturbome drug perturbation network. **a** The perturbome combines all 1832 identified interactions between 242 chemical compounds into a single network. Compounds resulting in strong morphological changes are colored in orange. Negative interactions are most frequent (red, 44%), followed by emergent (blue, 31%) and positive interactions (green, 21%). **b** Number of times that each of the 12 pairwise interaction types were observed. Most interactions are uni-directional, and bi-directional cross-talk is rare. **c** The degree-ordered adjacency matrix uncovers a pronounced core–periphery structure within the perturbome network. **d** The core consists mainly of negative interactions between drugs with strong morphotypes. **e** The majority of all observed interactions occur between the core and the periphery, often representing the modulation of a drug with a strong effect by a drug with a weak effect. **f** Interactions among drugs in the periphery are mainly emergent. **g** The degree distributions show the frequency of the number of neighbors per compound. There is a marked difference between the number of incoming and outgoing interactions. **h** Comparison of the number of observed interactions with randomized networks obtained from drug label randomization. Negative (positive) *z*-scores indicate that the observed number is smaller (larger) than expected by chance

We next extracted interaction profiles between the compounds using the vector-based methodology introduced above (see Methods section for details). In total, we identified 1832 interactions between 242 drugs that can be integrated into a single connected perturbome network (Fig. 4a). Given a total of 35,909 potential interactions, the observed connectivity of only 5% is remarkably sparse. Negative links are most frequent (44% of all edges), followed by emergent (31%) and positive (24%) links. The distribution over the 18 possible pairwise patterns shows a strong underrepresentation of reciprocal patterns (only 5% of all interactions), with mutual negative interaction being the most abundant of them (Fig. 4b). The identified perturbome thus indicates that in the vast majority of cases, two perturbations do not interfere with each other. In the relatively rare cases where they do, most commonly only one perturbation modulates the effect of the other.

The perturbome can be partitioned into a small core of densely connected nodes and a loosely interconnected periphery containing the majority of all nodes (Fig. 4c–f and Methods section)^31^. The core consists exclusively of strong perturbations, which are connected mainly by negative links (67%). This can be understood as a consequence of finite cell plasticity: physical constraints in attainable cell shape may prevent very strong morphological changes to be combined in a simple superposition, thus resulting in the observed negative interactions. Within the perturbome periphery, 85% of all interactions are emergent. This was also expected, as most of the respective perturbations (99%) show little morphological effects by themselves, hence any significant pairwise phenotype must be interpreted as emergent. Finally, the majority of all interactions in the perturbome are found between the core and the periphery, consisting of 46% negative, 29% positive, and 25% emergent links. Most interactions (75%) are directed from the periphery towards the core, thus representing the modulation of a dominant perturbation by a weaker one. This also explains the finding that hubs are observed only for incoming, but not for outgoing links (Fig. 4g).

We tested the robustness of the identified perturbome connectivity patterns by comparing it to randomized networks constructed using randomly swapped compound labels (see Fig. 4h and Methods section). The actual perturbome contains significantly fewer negative and emergent interactions (*z*-score = −57 and *z*-score = −46, respectively) and significantly more positive interactions (*z*-score = 29) than expected by chance. The higher number of negative interactions in random networks can be explained by random pairs where the two single perturbations contain morphological changes that are not present in the randomly chosen combination. Similarly, the higher number of emergent interactions results from morphological changes in the combination perturbation that are not present in the single perturbations. Since positive interactions can only occur when specific morphological changes of a single perturbation are amplified in the combination, they are less likely to occur by chance, thus resulting in the lower number observed in random networks. Taken together, these random controls confirm that the observed patterns cannot be attributed to general variability in cellular morphology, but are specific to particular perturbation pairs.

### Linking cellular, molecular, and pathophysiological features

**Fig. 5:**
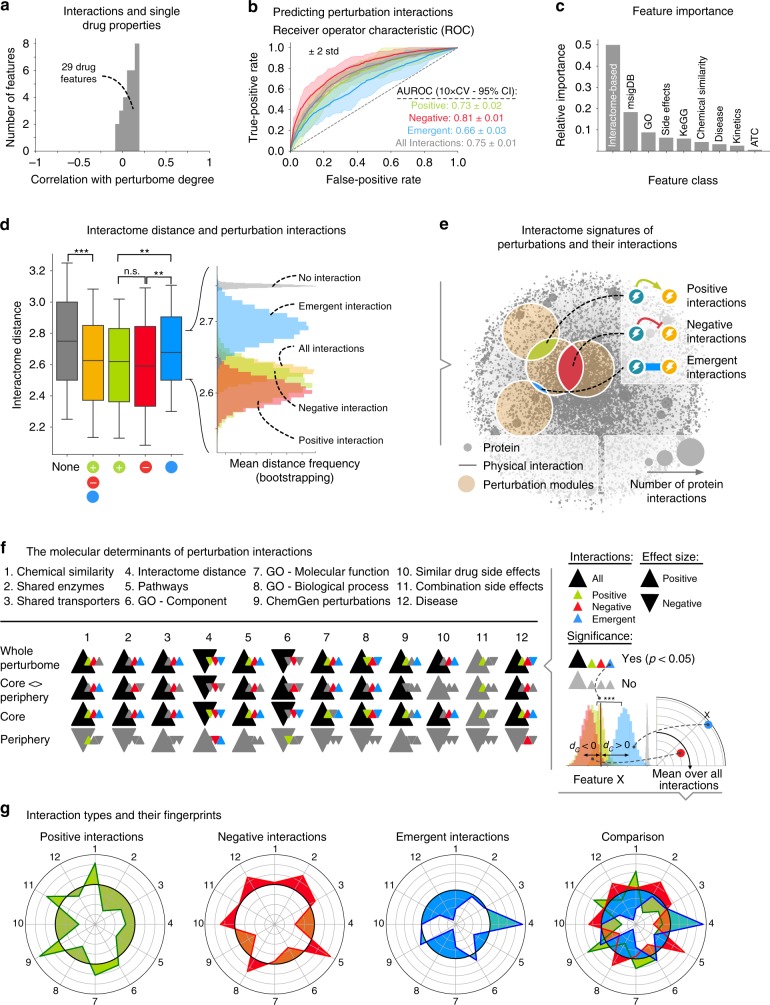
Linking cellular perturbation interactions with molecular and pathophysiological drug characteristics. **a** Individual drug characteristics do not correlate with the number of drug interactions. **b** Performance of a random forest machine learning classifier for predicting drug interactions from pairwise drug characteristics. **c** Relative importance of different feature classes for the predictions in **b**. **d** Distribution of interactome distances between the targets of two drugs that do not interact (gray) show any interaction (orange) and a positive (green), negative (red), or emergent (blue) interaction. The bootstrapping analyses confirms the significant differences among the respective means. The middle line in the boxplot displays the median, the box indicates the first and third quartile, whiskers the 1.5 interquartile range (IQR) (** and *** denote *P* values <0.01 and <0.001, Mann–Whitney *U* test). Outlier values are not displayed. **e** Interactome relationship between interacting perturbations: interacting drugs are generally characterized by overlapping modules. The extent of the overlap is predictive for the interaction type, from small overlap indicating emergent interactions to moderate and strong overlap indicating positive and negative interactions, respectively. **f** Summary of the relationships between the interactions observed on the cellular level and molecular or pathophysiological drug characteristics. Significant relationships with an interaction type are indicated by colored triangles that point up/down for enrichment/depletion compared to non-interacting drugs. **g** Interaction fingerprints highlighting how a given interaction type differs in its specific molecular and pathophysiological characteristics compared to the other interaction types. Numbers refer to the characteristics listed in **f**. Differences are quantified using Cohen’s *D*, *d*_C_, as a measure of effect size. The black central represents no difference (*d*_C_ = 0) in the respective interaction type relative to all interactions; gray lines indicate changes in increments of one unit of *d*_C_

We next investigated whether the interaction patterns observed on the cellular level can be associated with the molecular or pathophysiological levels (Supplementary Data 11). We collected a comprehensive set of 29 drug annotations (Supplementary Data 12 and Methods section), ranging from molecular information of the compounds (e.g., associated pathways, transporters, and metabolizing enzymes) to pathophysiological information (disease indications and known side effects). None of the individual annotations correlated strongly with the tendency of drugs to interact with others (Fig. 5a and Method section). In combination, however, they were predictive: we trained a random forest classifier to predict the observed drug–drug interactions based on a total of 67 drug pair features that were compiled from the 29 drug annotations introduced above and quantify the extent to which specific features are shared between two drugs (Supplementary Data 13 and Methods section). For the general presence of any drug interaction, the classifier reached an area under the Receiver Operating Characteristic (ROC) of AUROC = 0.74 ± 0.04 (mean ± 95% confidence interval, 10-fold cross validation, Fig. 5b). Interestingly, the performance differed significantly between interaction types: negative interactions are the most predictable (AUROC = 0.81 ± 0.2), emergent interactions the least (AUROC = 0.64 ± 0.02). Inspecting the contribution of different feature classes, we found that the interactome characteristics of the respective drug targets have by far the highest predictive power (Fig. 5c). All three interaction types are associated with interactome perturbations that are significantly closer than random expectation, yet we also observed clear differences among them: perturbations in very close interactome proximity to each other tend to result in negative interactions. Intermediate distances tend to result in positive interactions and, finally, emergence is associated with relatively distant interactome perturbations (Fig. 5d, e).

To further elucidate this finding, we next explored other significant relationships between the perturbome and molecular or pathophysiological drug characteristics. Figure 5f summarizes whether a given characteristic has a significant link to any interaction type (colored triangles) and whether it is enriched or depleted among interacting perturbations compared to non-interacting ones (triangle up/down). This analysis offers new insights, but also recapitulates several well-understood mechanisms of drug interactions. For example, chemically similar compounds may compete for the same target, thus altering the combined response relative to completely independent actions^32^ (Fig. 5f, column 1). The increased number of interactions among drugs with common transporters^33^ or metabolizing enzymes^34^ (Fig. 5f, columns 2 and 3) may be understood similarly: for example, a transporter (enzyme) that imports (metabolizes) a given drug under normal conditions may become unavailable when a second drug interferes with it. This would decrease the effective concentration of the former drug compared to the single perturbation, thus resulting in a negative interaction. Similar cases can be made for emergent or activating interactions. We further observed significantly increased functional similarity among the targets of interacting drugs in terms of shared pathways, GO annotations and transcriptional profiles (Fig. 5f, columns 5, 7–9). This is consistent with results obtained for genetic interactions in yeast^35^. We also find interesting enrichment patterns on the pathophysiological level: interacting drugs frequently share known side effects (Fig. 5f, column 10). The striking similarity with the enrichment patterns for common transporters and enzymes (compare Fig. 5f, columns 10 and 2 and 3, respectively) could suggest a direct link between side effect and a particular transporter/enzyme^36^. In contrast, only positive interactions are significantly enriched with drug interactions reported in the DrugBank database^37^ (Fig. 5f, column 11). In part, this may reflect the high number of reports describing an effective overdose of a particular drug when combined with another (70% of all interaction reports in DrugBank, see Supplementary Methods for an overview of the database). Finally, we find that our perturbation interactions are enriched for annotations to similar diseases (Fig. 5f, column 12). This follows from a combination of two interactome properties of drug-induced perturbations: first, drug perturbation modules typically overlap with the module of the respective disease they are used to treat^15,22^. Second, the drug interactions identified in our screen are strongly associated with an overlap of their respective perturbation modules.

Figure 5g condenses the commonalities and differences among the three interaction types in terms of molecular and pathophysiological fingerprints. For example, positive interactions are more strongly associated with chemical similarity than the other interaction types, whereas negative interactions have a unique peak for shared pathways, while emergent interactions are characterized by a distinctive spread on the interactome (Fig. 5g, green, red, and blue curves, respectively). Taken together, these discriminatory features provide a rich resource for further dissecting the different drug–drug interaction types.

## Discussion

The present study provides a mathematically founded strategy for investigating the combined effect of multiple perturbations with high-dimensional readouts. We showed that detailed cell-shape features can be used to quantify whether different drug perturbations either remain independent, influence each other in a directed positive or negative fashion, or result in the emergence of entirely unexpected phenotypic outcomes. Our analyses revealed distinctive molecular and pathophysiological fingerprints of the different interaction types. Most prominently, we found that the interactome-based distance between the targets of two perturbations determines their interaction: interacting perturbations tend to be co-localized in a common interactome neighborhood, whereas perturbations of distinct parts of the interactome tend to act independently. For perturbations within the same interactome neighborhood, the interaction type is determined by their proximity: close interactome proximity is associated with a decreasing effect, medium interactome distances with increasing effects, whereas entirely unexpected phenotypic outcomes emerge at the boundaries of the respective perturbation neighborhoods.

These findings complement previous studies showing that diseases whose associated genes reside in overlapping interactome neighborhoods are characterized by increased clinical similarity and comorbidity compared to diseases affecting separate interactome neighborhoods^13^. Taken together, this suggests a generalized view according to which both therapeutic and disease associated perturbations can be understood as localized interactome perturbations whose interactions are determined by their interactome overlap. The striking ability of the interactome to isolate the impact of separate perturbations despite the highly connected nature of the underlying molecular network is also reflected in the overall sparseness of the perturbome identified in this study.

The methodologies introduced here can also be applied to other types of high-dimensional readouts, such as transcriptional profiling, or other types of perturbations, for example in genetic interaction studies^25,38^ or studies of dosage dependent drug effects (see Supplementary Methods, Supplementary Data 14 and Supplementary Fig. 11 for a basic proof-of-concept application of morphological effects across drug concentrations). Targeted perturbation has long been the prime tool for elucidating the function of individual components of a biological system. More recently, also pairwise perturbations have been investigated at an increasing scale in both healthy and disease conditions^39,40^. To facilitate the application of our methodology to these or other studies, the computational pipelines used in this work are available within the Supplementary Material. We further provide the raw imaging data, as well as the extracted perturbome network as a resource for more advanced analyses, for example to investigate connection patterns involving multiple link types and more than two perturbations using graphlet approaches^41^.

## Methods

### Interactome construction

For the construction of the human interactome, we used the Human Integrated Protein Protein Interaction Reference (HIPPIE) database (version 2.1, see Supplementary Methods for more details of the database)^42,43^, which contains 322,599 confidence scored and functionally annotated interactions between 17,053 human proteins and lncRNAs. Filtering for proteins and interactions with at least one literature reference results in a PPI network with 16,393 nodes and 309,365 links (Supplementary Data 1). We restrict our analysis to the largest connected component containing 16,376 nodes and 309,355 links. The interactome is approximately scale-free and shows other typical characteristics observed previously in many biological networks^44^, such as high clustering and short path lengths (Supplementary Fig. 1).

### Drug library and target annotation

We used the previously established CeMM library of unique drugs (CLOUD) that was designed to capture as much as possible of the chemical and biological diversity of all US Food and Drug Administration (FDA) approved drugs^26^ (see Supplementary Fig. 2 and Supplementary Methods for a characterization of the CLOUD library). The library consists of 314 drugs, of which 267 have been used in this study (Supplementary Data 2). We extracted targets for all drugs from three databases: (i) DrugBank^45^, (ii) PubChem^46^, and (iii) ChEMBL^47^ (see Supplementary Data 3 for the various identifiers). For DrugBank, we manually downloaded the complete dataset from [https://www.drugbank.ca/releases/latest, Sept. 2018] and then parsed the xml file using a custom Python script. As targets, we included all gene associations from various DrugBank sections, namely targets, transporter, enzymes, and carriers. For PubChem and ChEMBL, we used the REST-based API (application programming interface) to download drug information. The PubChem database contains a direct qualifier for active targets. For ChEMBL, we included experiments with IC_50_, *K*_i_, or EC_50_ and used a typical 10 μM cutoff. We then used DrugBank to filter out annotations that represent enzymes and transporters instead of direct targets, e.g., the *CYP* genes, resulting in the “Target filtered” list that we used for our analysis (compare with Fig. 1b, red circles, and Supplementary Fig. 3 and Data 5).

### Interactome characterization of the drug targets

We characterize drug targets on the interactome using several network-based measures summarized in Supplementary Fig. 4: (i) The degree *k* of an individual protein, giving the number of its neighbors within the interactome. (ii) The centrality *c*, quantifying the fraction of all pairwise shortest paths that pass through a node. (iii) The size *S* of the largest connected subgraph formed by a given set of nodes. (iv) The average shortest distance 〈*d*_s_〉: for each of the *N*_*d*_ target proteins, we determine the distance *d*_s_ to the next-closest protein associated with the same drug. The average 〈*d*_s_〉 can be interpreted as the diameter of a perturbation module on the interactome. We compared this average to mean distance $\mu _{\mathrm{{random}}}$ (with respective standard deviation $\sigma _{\mathrm{{random}}}$) obtained from 10k random modules of the same size and quantified the localization of the original protein set using Glass Δ as effect size:

1 $${{\Delta }} = \frac{{\left\langle {d_{\mathrm{s}}} \right\rangle - \mu _{\mathrm{{random}}}}}{{\sigma _{\mathrm{{random}}}}}.$$

(v) The network-based overlap *s*_*AB*_ between two drugs *A* and *B* is measured by comparing the diameters 〈*d*_*AA*_〉 and 〈*d*_*BB*_〉 of the respective modules of the individual drug targets to the mean shortest distance 〈*d*_*AB*_〉 between them:

2 $$s_{AB} = \langle d_{AB}\rangle -(\langle d_{AA}\rangle + \langle d_{BB}\rangle )/2.$$

Positive values of *s*_*AB*_ indicate that the two drug modules are separated on the interactome, whereas negative values correspond to overlapping modules^13^. (vi) The mean distance 〈*d*_Mean*AB*_〉 of all pairs of targets between the two drugs, which can be interpreted as a proximity measure between the two respective drug modules^44^.

### Quantifying biological similarity of proteins

We quantified the biological similarity of proteins according to their annotated (i) GO terms (ii) disease and (iii) side effect (SE) associations. GO annotations^48^ were extracted from [http://www.geneontology.org/, downloaded Aug. 2018]. We removed annotations associated with the evidence codes IEA (inferred from electronic annotation), ND (no biological data available), and IPI (inferred from physical interaction) in order to avoid circularity in the interactome-based evaluation of the similarity of proteins. The filtering reduces the number of annotations by 38% (from 476k to 296k annotations).

To construct a comprehensive set of gene-disease annotations, we combined information from the disease ontology extracted from [http://disease-ontology.org/, downloaded April 2018] with gene–disease associations from [http://www.disgenet.org/, downloaded November 2018]. We included only the following sources (in decreasing order of the respective number of contained diseases): HPO^49^, CTD^50^, PSYGENET^51^, ORPHANET^52^, UNIPROT^53^, resulting in 130k gene–disease associations. Disease annotations were mapped between the two databases using the Unified Medical Language System (UMLS)^54^ obtained from [http://www.disgenet.org/web/DisGeNET/menu/downloads, November 2018]. In total, we could map approximately 69% of all annotations (ca. 90k out of 130k), resulting in 7795 genes linked to 3630 diseases (Supplementary Data 15).

Side effects were extracted from the Offsides database^55^ [http://tatonettilab.org/resources/tatonetti-stm.html, April 2018]. In total, we identified 7685 unique side effects annotated to a CLOUD drug, on average, each drug was annotated to 269 side effects. Anatomical Therapeutic Chemical Classification System (ATC) classes for the individual drugs were extracted from DrugBank [https://www.drugbank.ca, downloaded April, 2018].

For the GO and disease ontology we used a similarity metric based on the information content of the individual terms within the respective ontology trees: functional similarity between drug protein targets is quantified by the specificity of their shared annotations, assuming that proteins sharing very specific functions are more similar to each other than those who only share generic annotations^8^. The specificity of a term *i* is measured by the total number of proteins *n*_*i*_ annotated to it. The similarity *S(a, b)* of two proteins *a* and *b* is then determined by the most specific term they share:

3 $$S(a,b) \equiv \frac{2}{{\mathrm{{min}}(n_i)}}$$

The value of *S*(*a*, *b*) ranges from *S*(*a*, *b*) ≡ 0 for no shared terms, to *S*(*a*, *b*) = 1 if *a* and *b* are the only two proteins annotated to a specific GO term. The overall functional similarity of a set of proteins associated with a particular drug is measured by the average *S(a, b)* over all *n*_pairs_ pairs of drug-associated proteins:

4 $$\left\langle S \right\rangle = \frac{1}{{n_{\mathrm{{pairs}}}}}{\sum} {S(a,b)}.$$

Since the side effects extracted from the Offsides database are not ontology based, we use the amount of overlapping side effects to quantify similarity.

### Identification of interactions between perturbation

To calculate effect and directionality of the interactions between two perturbations we treat both the single perturbations, as well as the double perturbation as vectors pointing from the unperturbed state to the perturbed one within an *n*-dimensional feature space. Here, we use morphological features that describe the shape of cells to define the feature space (Supplementary Fig. 6A–C). Note, however, that our framework is applicable to any high-dimensional readout. We used the following steps to define and calculate interactions: the distance between two points $\vec x$ and $\vec y$ with coordinates $\vec x = (x_1,x_2,...,x_n)$ and $\vec y = (y_1,y_2,...,y_n)$ in an *n*-dimensional space is given by

5 $$d^2 = \mathop {\sum }\limits_{i = 0}^n (x_i - y_i)^2.$$

In our case, we have the two single treatments *A* and *B*, as well as the combination treatment *C*, whose corresponding vectors we denote as $\vec a$, $\vec b$, and $\vec c$, respectively. The vectors $\vec a$ and $\vec b$ span a two-dimensional surface *S* that also contains the origin:

6 $$S:\vec x = r\vec a + s\vec b.$$

The distance of the point $\vec c$ from this surface thus reads

7 $$d^2(r,s) = \mathop {\sum }\limits_{i = 0}^n (ra_i + sb_i - c_i)^2.$$

Minimizing the distance yields a vector pointing perpendicular from the surface to the point $\vec c.$ Setting the partial derivatives of the distance with respect to *r* and *s* to zero results in the matrix equation

8 $$A\;\vec p = \vec h,$$

where the matrix *A* is given by $$A = \left( {\begin{array}{*{20}{c}} {a^2} & {\vec a.\vec b} \\ {\vec a.\vec b} & {b^2} \end{array}} \right)$$, and with the vectors $\vec p = \frac{r}{s}$ and $$\vec h = \left( {\begin{array}{*{20}{c}} {\vec a.\vec c} \\ {\vec b.\vec c} \end{array}} \right)$$.

The parameters *r* and *s* can be calculated by inverting the matrix *A* via

9 $$\vec p = A^{ - 1}\vec h.$$

Inserting the resulting *r* and *s* in the surface Eq. (6) gives the projection point from $\vec c$ onto the surface *S*. The orthogonal vector then reads $\vec n = \vec x_{r,s} - \vec p$ and holds $\vec n \ast \vec a = 0$ and $\vec n \ast \vec b = 0$ . Plugging *r* and *s* into Eq. (7) finally yields the minimal distance. We can now decompose every possible combination vector $\vec c$ into two components within the 2D surface *S* and a third component pointing outward:

10 $$\vec c = \alpha \vec a + \beta \vec b + \gamma \vec n.$$

Non-interaction between two perturbations can be identified with a combination vector that represents exactly the superposition of the two single ones, i.e., $\alpha = \beta = 1$ and $\gamma = 0.$ Any deviation from this superposition hence indicates an interaction between perturbations. The different permutation of *α* and *β* being equal to/smaller/larger than one, as well as *γ* being zero or not, divide the whole state space into a total of 27 subspaces (1 point, 6 1D lines, 12 2D surfaces, and 8 3D spaces), such that any combination vector can be assigned to exactly one of them (Supplementary Fig. 6D). As both positive and negative values of *γ* map to the same emergent interaction type, there are only 18 subspaces that correspond to different interaction types and which are depicted in Fig. 1e and Supplementary Fig. 6E. Note that in principle, the absolute values of *α*, *β*, and *γ* could be used to quantify the magnitude of an interaction. In this work, however, we considered an unweighted network in order to investigate the basic qualitative properties of the different interaction types. To this end, we applied very stringent statistical criteria for the presence or absence of an interaction, see below for a detailed discussion of the construction of the perturbome.

### High-throughput high-content imaging screen

We used MCF-10A (Michigan Cancer Foundation-10A) cells for our screen, an adherent, non-tumorigenic breast epithelial cell line that exhibits a rich morphology and has been used previously in high-content imaging screens^18,56^. MCF-10A cells arose without exposure to chemical or viral carcinogens, exhibit only minimal changes to the genome, do not show any characteristics of invasiveness, and also do not form tumors when transplanted into immunodeficient mice^57,58^. Drugs were transferred on 384-well plates using an acoustic liquid handler capable of transferring smallest amounts of liquids (2.5 nL). The cells were then seeded on top of the drugs using an automatic cell dispenser and incubated at 37 °C for 72 h and 5% CO_2_ in DMEM-F12 cell media (Supplementary Data 6). For fluorescence staining, the medium was removed and living cells were first stained for mitochondria using MitoTracker Orange (1 µM in 1× PBS, 30 min at 37 °C). Next, the cells were 3× washed with PBS and fixed with cold methanol at −20 °C overnight. Methanol was removed and 3% BSA and 0.1% Triton-X in PBS were added for permeabilization/blocking (2 h at room temperature, RT). The blocking solution was removed and the cells were incubated with the first antibody (anti-Tubulin, 1:500 in 3% BSA, 0.1% Triton-X in PBS) overnight at 4 °C in the cold room. The following day, the Alexa Fluor 488 secondary antibody (1:1000) for visualizing the cytoskeleton and 4′,6-diamidin-2-phenylindol/DAPI (1:500) for staining the DNA in 3% BSA, 0.1% Triton-X in PBS were added (2 h at RT). In a final step, cells were washed 3× with PBS. Four images (foci) (20×) (see Supplementary Fig. 12 for example images) per well were acquired in a high-throughput manner using the Operetta High-Content Imaging System (PerkinElmer) in three fluorescent channels, DAPI (405/470 nm), BetaTubulin (488/525 nm), and MitoTracker (554/576 nm). The whole screen was performed in two batches.

### Image analysis and feature extraction

We used the software CellProfiler (version 3.0.0)^29,59^ to process the images from the screen, see Supplementary Fig. 13 for an overview of our feature extraction pipeline, which is also provided as Supplementary Data 7. Some parameters of the pipeline were adjusted between the two batches of the screen, e.g., minimum intensity thresholds for segmentation to account for slight differences, such as different exposure times. The pipeline first corrected the image channels for uneven illumination before performing image segmentation and feature measurements. We next applied an image quality workflow to exclude images that are, e.g., out of focus or have problems with saturation. The quality control workflow used 45 quality image measurements provided by CellProfiler (Supplementary Data 8), in combination with an isolation forest method to detect outliers as implemented in the Python package Scikit-learn (v0.2.1)^60^. Isolation forest is an ensemble method based on decision trees. Since outliers are less frequent than regular observations, they can be detected by being closer to the root of decision trees, i.e., they require less splits to be uniquely characterized. We identified around 3% of all images (ca. 6000) as outliers. For our subsequent analysis, we included only wells with at least three out of four valid images.

In total, we calculated 438 cell morphology features, which include descriptors of geometry, intensity distributions, texture, as well as adjacency statistics measured for the cell, nuclei, and cytoplasm (Supplementary Data 8). The features were normalized as follows: we first calculated the median of the cellular features over all cells in a given well. It has been shown previously that small-molecule effects are well characterized by the shift of measures-of-centers (mean or median) of their phenotypic response^30,61^. Next, we performed a Tukey’s median polish to reduce the impact of plate effects, such as evaporation towards the borders of a plate. This procedure fits an additive model for data in a two-way layout table of the form row effect + column effect + overall median^62^. Finally, we scale all values to the range between 0 and 1, considering values outside the 99.5/0.05 percentiles as outliers.

### Feature filtering

To include only robust and informative features, we applied the following filtering pipeline: (i) For each feature, we calculate the coefficient of variation (CV) among the DMSO controls of the same plate. The coefficient of variation is a stable measure for the variation among samples normalized by their mean. The CV is calculated both within replicates on the same plate (intra-plate CV), as well as between plates (inter-plate CV). Only features whose inter- and intra-plate CV are smaller than 0.2 were included in the further analysis. (ii) We removed features that show too little correlation between drug treatment replicates (cutoff: Pearson’s *ρ* < 0.2). (iii) We evaluated the effect size of each feature *z*-scores, and removed all features without significant effect in any single or combinatorial perturbation by transforming the *z*-scores to *P* values and applying a cutoff of *P* value <0.05 after Bonferroni correction for multiple hypotheses. (iv) Finally, we removed redundant features: we first calculated all pairwise feature correlations. For any feature pair with a correlation of *ρ* > 0.8 we then removed the one with the smaller effect size using an iterative minimal cut approach (Supplementary Fig. 14). This step further ensures approximate orthogonality for the vector-based framework introduced above. Steps (i) through (iii) are applied separately to the two batches. At step (iv), all features that fulfilled all requirements for both batches before removing correlating features were included. In total, 78 out of 438 (17.8%) features were included for the subsequent analyses (Supplementary Data 9).

### Identification of significant perturbations

We first filtered the drug perturbations for various aspects: (*i*) We removed all wells with technical issues during the drug transfer, e.g., compound precipitation. (ii) We excluded wells with less than 30 cells (Supplementary Fig. 7A). (iii) All single drug perturbations with less than three valid wells (replicates) were excluded. (iv) We tested the stability of each drug, i.e., whether its activity decreased over the course of the whole screen, by comparing the six individual replicates that were plated at different time points from the same source plate. Drugs that lose activity over time should exhibit a lower cytotoxicity at the later replicates. We defined stability using a linear regression over the individual time points:

11 $$f(x) = a + bx.$$

Drugs with *b* > 0.05 and a difference of at least 30% between the well with the largest cell count and lowest cell count were excluded from the further analysis (Supplementary Fig. 7B). Out of the initial 267 drug perturbations, 242 fulfilled all criteria and were further used in our morphological analysis and perturbation interaction calculations (Supplementary Data 10). To distinguish significant perturbations from random fluctuations, we used the Mahalanobis distance to quantify the extent of morphological changes induced by a given perturbation. The perturbation Mahalanobis distance *D*_P_ can be understood as a multi-dimensional generalization of the *z*-score and measures by how many standard deviations a vector $\vec x$ differs from a distribution *D* with mean $\vec u$ and covariance matrix S:

12 $$D_P(\vec x) = \sqrt {(\vec x - \vec u)^{{T}}S^{ - 1}(\vec x - \vec u)}.$$

To reduce noise, we applied a PCA to the original feature space before calculating the Mahalanobis distances^27^, such that the considered principal components collectively explain 90% of all variance. We used a cutoff of *D*_P_ > 7 to distinguish significant perturbations from non-significant. Overall, 28 out of 242 drug perturbations (12%) met this criterion in at least one of the two batches (Supplementary Fig. 7C, D) and were considered as strong perturbations in the subsequent analyses (compare with Figs. 3c and 4). For the single drug perturbations we calculated the mean vector for the corresponding replicates and compared it to the DMSO vectors across the whole batch. For the combination perturbations, we used the single measurement and compared it to the DMSO vectors across the whole batch.

### Quantifying similarity between feature vectors

We used the cosine similarity *S*_cos_ that quantifies the similarity between two vectors $\vec A$ and $\vec B$ in an *n*-dimensional space via the angel $\theta$ between them:

13 $$S_{\cos} = \cos(\theta ) = \frac{{\vec A \ast \vec B}}{{||A||\;\;||B||}} = \frac{{\mathop {\sum }\nolimits_{i = 1}^n A_iB_i}}{{\sqrt {\mathop {\sum }\nolimits_{i = 1}^n A_i^2} \sqrt {\mathop {\sum }\nolimits_{i = 1}^n B_i^2} }}.$$

The resulting similarity values range from *S*_cos_ = −1 for vectors pointing in opposite directions, to *S*_cos_ = 1 for vectors with the same orientation, with *S*_cos_ = 0 indicating orthogonality and values in-between indicating intermediate (dis)similarity. Note that the cosine similarity only considers the orientation of two vectors, so that different magnitudes of the same phenotype are considered the same using this measure. We thus included an additional filter for small non-significant perturbations that are merely random effects. Using a vector length at least two standard deviations away from the mean vector norm of all DMSO wells (Supplementary Fig. 10A) we assessed the similarity of cells treated with (i) the exact same drug (but different replicates), (ii) drugs with the same MOA, (iii) drugs with same ATC code, and (iv) drugs with similar interactome distance (Supplementary Fig. 10 and Data 4).

### Constructing the drug perturbation interaction network

We assessed the significance of an interaction using the distance of the measured combination vector from the non-interaction point defined by the sum of the individual vectors. To incorporate the variability of the individual drug perturbations, we calculated all possible pairwise vector sums for all corresponding single drug replicates (Supplementary Fig. 15A). Next, we included the intra-plate variance, i.e., how much cells fluctuate between wells on a plate, by multiplying each calculated vector sum with the corresponding DMSO controls of the same plate as the combination perturbation (Supplementary Fig. 15B). The resulting point cloud reflects the high-dimensional space in which the combination vector is expected to fall for non-interacting perturbations. By calculating the Mahalanobis distance between the measured combination vector and this non-interaction space we can calculate how likely the combination effect arose from a true effect that cannot be explained by random fluctuations (Supplementary Fig. 15C). We considered combinations with an interaction Mahalanobis distance *D*_I > _3 as indicative of a true interaction. In addition to the significance, we also included a measure of effect size. We calculated drug specific thresholds using the distributions of α, β, and γ values for interactions that are not significant, i.e., that have D_I_ ≤ 3. For α, β, we set the effect size threshold to be at least 2 median absolute deviations (MAD) away from the respective medians of all non-significant drug pairs (Supplementary Fig. 15D). For γ, we used the largest γ found among non-significant pairs as threshold (Supplementary Fig. 15E). This is to prevent cases, where already small deviations in the combination might be of nominal significance due to single drug perturbations with only small effects, and hence also small variances, that may result in misleadingly large Mahalanobis distances.

### Characterization of the perturbome network

To assess the core-periphery structure within the perturbome network we used the Python package cpalgorithm (version 0.0.14)^63^. The package contains an implementation of the MINRES (minimal residual) algorithm^64^, which assigns each node in the perturbome to either the core or the periphery based on a singular value decomposition (SVD) of the adjacency matrix of the network. The statistical significance of the resulting assignment was tested using the (*q*,*s*)-test^65^, which calculates a *P* value based on a comparison of the core–periphery structure within the perturbome to the core–periphery structure of 1000 randomized networks created using the configuration model.

To calculate the sparseness of the perturbome, we first determined the total number of possible edges: the network consists of two node types, representing drugs with strong (*s*) or weak (*w*) phenotype, as well as directed (positive/negative) and undirected (emergent) edges. Only a single emergent interaction is possible between two drugs with weak phenotypes, two interactions are possible between one drug with weak and one with a strong phenotype, and three interactions between two drugs with a strong phenotype. Therefore, the total number *M*_max_ of possible interactions can be calculated as

14 $$M_{\mathrm{{max}}} = \frac{{w(w - 1)}}{2} + 2ws + 3\frac{{s(s - 1)}}{2}$$

with *s* = 28 and *w* = 214, the possible number of links in the perturbome is given by *M*_max_ = 35,909. We also applied this formula to calculate the number of possible links within the core and the periphery, as well as between them. The sparseness is finally determined by the ratio of observed and maximally possible number of links (compare with Fig. 4a, d–f).

We assessed the robustness of the perturbome and the specificity of its edges by comparing it to 10,000 randomized perturbomes of same size. Random perturbomes were created by switching the labels of the individual drug perturbation so that a combination vector is always compared to single vectors that are not part of the combination. For example, a combination vector AB would be compared to the two single vectors C and D. A single random perturbome then results from testing the same number of interactions as in the original perturbome, using the same parameters and thresholds as introduced above.

### Linking interactions with drug characteristics

We used the data sources described in the Supplementary Methods (Supplementary Data 12) to calculate the following features for characterizing individual drugs and drug pairs: (i) Chemical similarity as defined by the Tanimoto coefficient between two Molecular Access System (MACCS) structural fingerprints. The MACCS fingerprints were created using the SMILE strings (Simplified Molecular-Input Line-Entry System) of the respective molecules. Conversion and calculations were performed using the Python package RDKit [version 2018.09.01, https://www.rdkit.org/docs/GettingStartedInPython.html]. (ii) Interactome distance was characterized using the three measures 〈*d*_*AB*_〉, 〈*d*_Mean*AB*_〉 and 〈*d*_Min*AB*_〉 on all five drug target sets introduced above (DrugBank, ChEMBL, PubChem, Combined, Target Filtered). (iii) For features that quantify the overlap between two drugs, e.g., the number of shared pathways or GO annotations, we used both the absolute number of common annotations, as well as a normalized version divided by the union of both annotations. (iv) Features that quantify the biological similarity were calculated as described in the respective section above. Overall, we calculated 29 features for single drugs (Supplementary Data 12), and 67 features for drug pairs (Supplementary Data 13).

To analyze whether any of the 29 collected drug characteristics correlates with the general tendency of a drug to interact with others (compare with Fig. 5a), we calculated the Pearson correlation coefficient for each drug feature *F* and the total number of interactions per drug, i.e. its degree *k*. The 67 drug pair features were analyzed using a random forest classifier from the Python package Scikit-learn (v0.2.1)^60^ (parameters: max_depth = 25, estimators = 150, class_weight = “balanced”, criterion = “gini”, max_features = “auto”). We calculated the area under the receiver operator characteristic (AUROC) for each interaction type (positive, negative, emergent) separately, as well as for all interaction types combined, using a 10-fold cross validation procedure (Fig. 5b). We also calculated the standard deviation for each point at the ROC curve. Random forest classifiers allow for an inspection of the relative importance of the 67 individual features by considering the number of times a feature was picked as a split among the decision trees constructed by the classifier. We summarized the 67 features into 9 groups based on their respective type and data source (Fig. 5c).

The bootstrapping procedure shown in Fig. 5d for quantifying the robustness of the individual means and further characterizing the differences between the different interaction types was performed as follows: we randomly sampled *n* values from the original distribution (*n* = number of values in the distribution) with replacement. This was performed 10,000 times and each time the mean was calculated, resulting in a distribution of means.

The association between individual features and a given interaction type (see Fig. 5f) was quantified using either using Fisher’s exact test (for binary features, i.e. those that either overlap or not) or the Mann–Whitney *U* test (for all other features). We calculated *P* values, as well as odds-ratios or fold-changes between the individual groups (indicated as direction of the triangle in Fig. 5f). As cutoff for significant features, we used a *P* value of ≤0.05 (indicated as colored triangles in Fig. 5f). To quantify the differences among the interaction types, we calculated Cohen’s *d* between each individual interaction type (positive, negative, and emergent) compared to all interactions. Cohen’s *d* is defined as

15 $$d = \frac{{\mu _1 - \mu _2}}{s}$$

with the pooled standard deviation

16 $$s = \sqrt {\frac{{(n_1 - 1)s_1^2 + (n_2 - 1)s_2^2}}{{n_1 + n_2 - 2}}}$$

### Reporting summary

Further information on research design is available in the Nature Research Reporting Summary linked to this article.

## Supplementary information

Supplementary Information

Description of Additional Supplementary Files

Supplementary Data 1

Supplementary Data 2

Supplementary Data 3

Supplementary Data 4

Supplementary Data 5

Supplementary Data 6

Supplementary Data 7

Supplementary Data 8

Supplementary Data 9

Supplementary Data 10

Supplementary Data 11

Supplementary Data 12

Supplementary Data 13

Supplementary Data 14

Supplementary Data 15

Reporting Summary

## Data Availability

All data used in this study is freely available as a supplement to this manuscript (Supplementary Data). The raw imaging data produced and analyzed in this study were deposited to the Image Data Resource (https://idr.openmicroscopy.org) under accession number idr0069 (ref. ^66^). The perturbome network can be inspected interactively and downloaded from the NDEx platform^67^ under https://tinyurl.com/y22ep2em

## Code availability

Computer code is available from GitHub under https://github.com/menchelab/Perturbome.
