# Supplementary material for: Mapping the perturbome network of cellular perturbations
Source: Nat Commun. 2019 Nov 13;10:5140. doi: 10.1038/s41467-019-13058-9 (PMC6853941; doi:10.1038/s41467-019-13058-9)
Supplement: Supplementary file 1 — Supplementary Information [file 41467_2019_13058_MOESM1_ESM.pdf]

Supplementary Information for

Mapping the perturbome network of cellular perturbations

Michael Caldera[1], Felix Müller[1], Isabel Kaltenbrunner[1], Marco P. Licciardello[1,2], Charles-Hugues Lardeau[1,3], Stefan Kubicek[1] & Jörg Menche[1,*]

* Corresponding author. E-mail: jmenche@cemm.oeaw.ac.at

The PDF file includes:

Supplementary Figures 1
Supplementary Figure 1: Human Interactome characteristics. 1
Supplementary Figure 2: The CLOUD library. 2
Supplementary Figure 3: Combining several drug annotation databases. 3
Supplementary Figure 4: Network characteristics and distance measurements. 4
Supplementary Figure 5: Drug module localization and biological characteristics. 5
Supplementary Figure 6: Deriving perturbation interactions from cell morphology changes. 6
Supplementary Figure 7: Identifying significant perturbations. 7
Supplementary Figure 8: The heterogeneity of cellular response to perturbations. 8
Supplementary Figure 9: Cosine distance and visual similarity. 9
Supplementary Figure 10: Connecting morphological and biological similarity. 10
Supplementary Figure 11: Morphological profiles across different drug concentrations. 11
Supplementary Figure 12: Example images for MCF-10A cells treated with cytarabine. 13
Supplementary Figure 13: Overview of the image analysis and feature extraction pipeline. 14
Supplementary Figure 14: Network-based approach for removing correlating features. 15
Supplementary Figure 15: Calculating the interaction thresholds. 16
Supplementary Methods 17
CLOUD library characterization 17
Investigating cell population heterogeneity 17
Comparing feature vector similarity and visual similarity 19
Overview databases 19
Morphological effects across drug concentrations 22
Supplementary References 25

Supplementary Figures

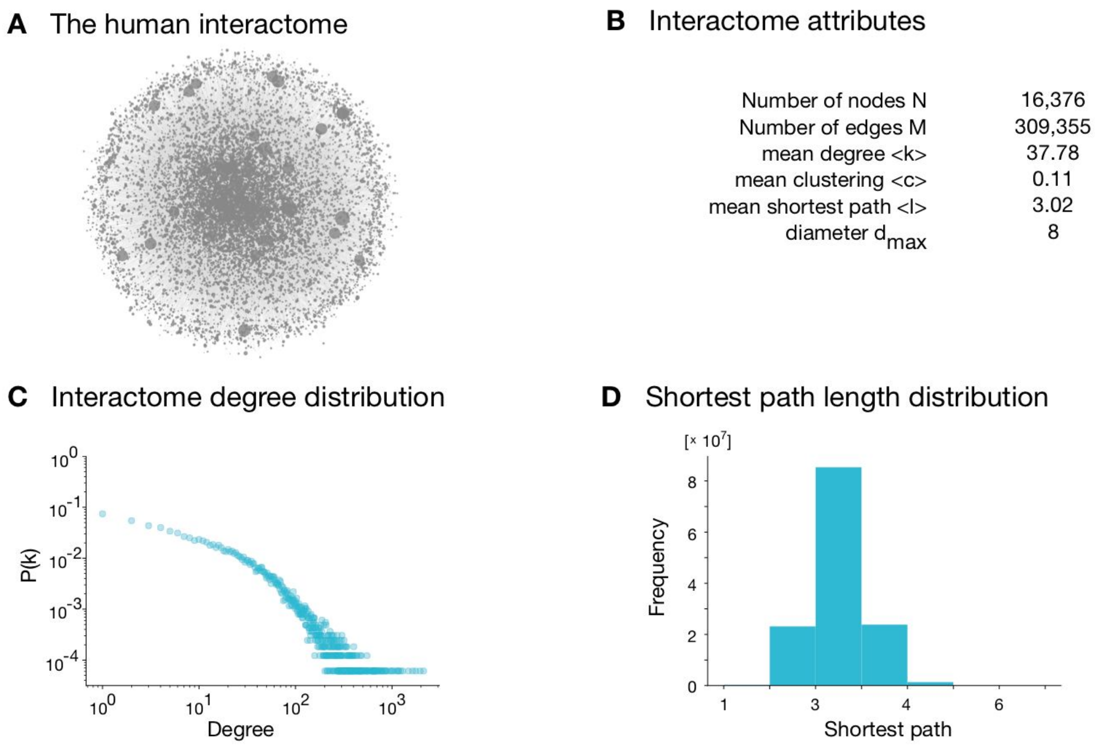

Supplementary Figure 1: Human Interactome characteristics.

(**A**) Visualization of the interactome with 16,376 proteins and 309,355 physical interactions between them. Node size reflects its degree *k*. (**B**) Basic network characteristics. (**C**) Degree distribution *P*(*k*). (**D**) Shortest path length distribution with mean $\langle l \rangle$ = 3.02, i.e. the vast majority of proteins in the human interactome are connected to each other within 3 steps.

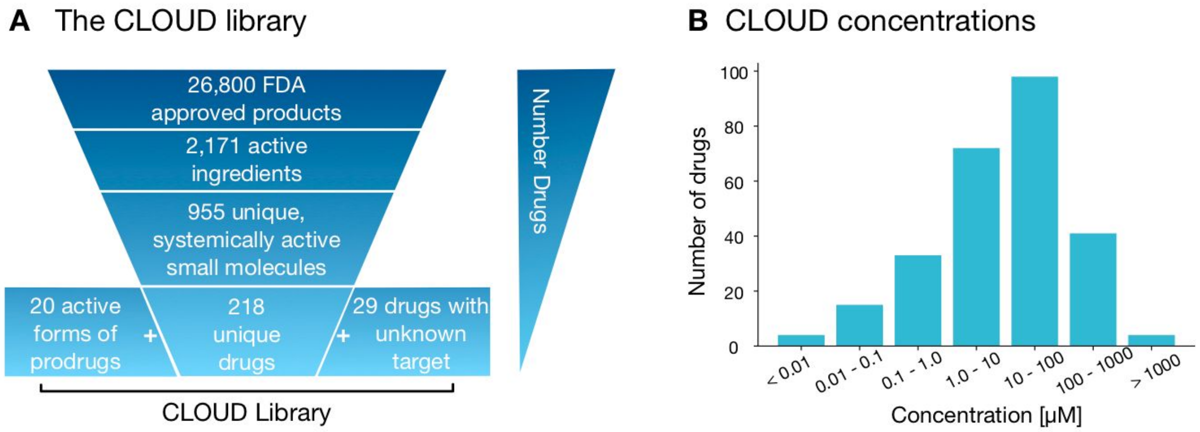

Supplementary Figure 2: The CLOUD library.

(**A**) Overview of the filtering and data mining steps starting from all FDA approved products to the final CLOUD (CeMM Library Of Unique Drugs) drug library, which contains 267 drugs representing most drug and target classes. (**B**) Concentrations used across all CLOUD drugs.

A Database overview

	DrugBank	PubChem	ChEMBL	Combined	Target Filtered
Total amount of targets:	2230	3569	1005	5365	3643
Amout of distinct targets:	529	990	350	1241	1096
Mean targets:	8.35	13.37	3.76	20.09	13.64
Median targets:	6	6	1	12	6

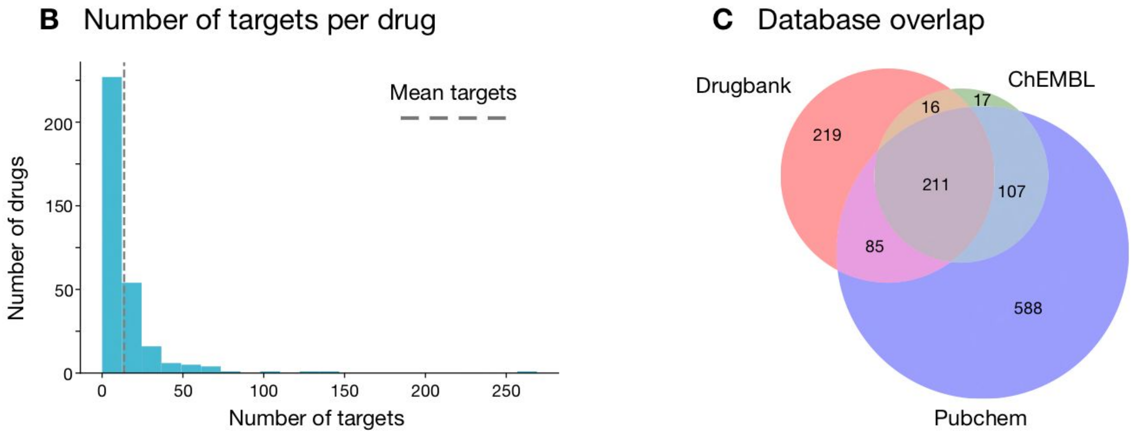

Supplementary Figure 3: Combining several drug annotation databases.

Overview of the different drug-protein annotation sources that have been used: (**A**) Number of annotations extracted from DrugBank, PubChem and ChEMBL, as well as their combination ('Combined') and filtered for molecular targets only ('Target Filtered'). (**B**) Number of filtered targets per CLOUD drug. (**C**) Overlap between the three drug protein annotation sources.

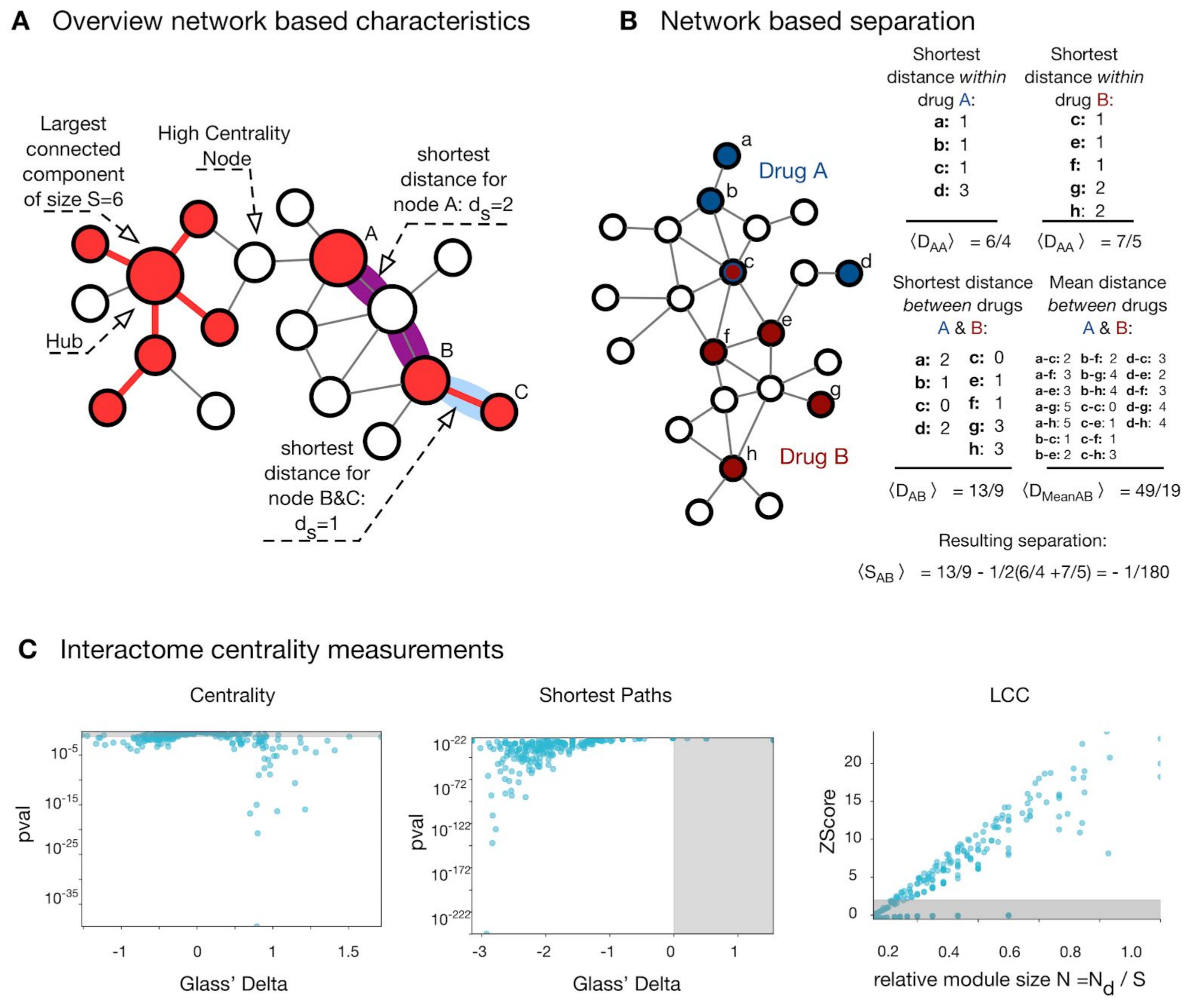

Supplementary Figure 4: Network characteristics and distance measurements.

(**A**) Illustration of various network characteristics. Red nodes represent drug targets and thick lines shortest distances between two nodes. Connected subgraphs of targets are indicated by red lines. (**B**) Illustration and example calculations for different network-based distance measures. (**C**) Centrality and localization of CLOUD protein targets within the interactome. The relative module size *N* is calculated by dividing the largest connected component size *S* by the total number of all annotated proteins N_d.

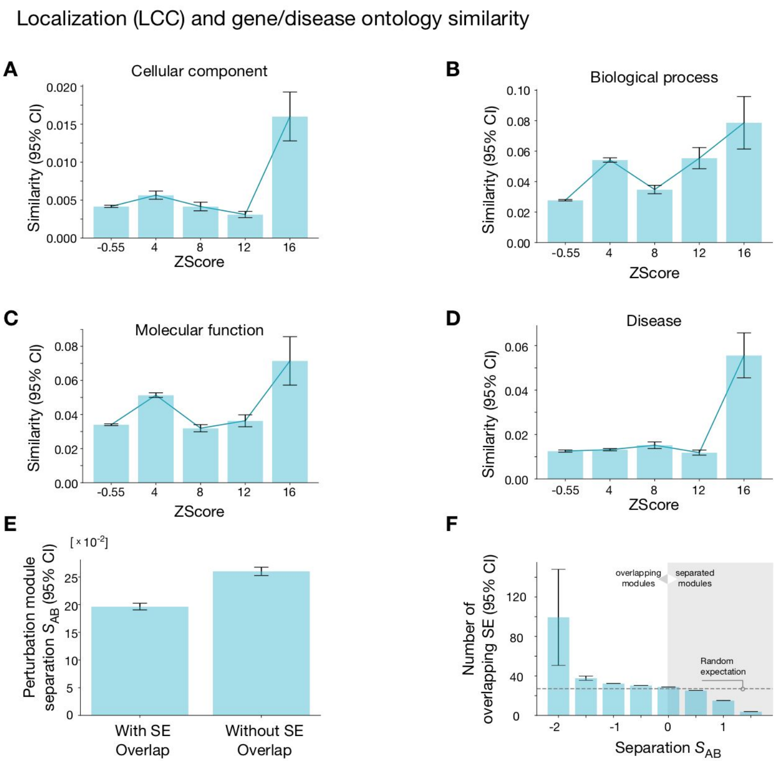

Supplementary Figure 5: Drug module localization and biological characteristics.

(**A** to **D**) Drug modules with higher interactome localization are characterized by higher similarity of their respective targets in terms of GO term annotations of (A) cellular component, (B) biological process, (C) molecular function, as well as in terms of (D) annotated diseases. Drug module localization is quantified by the *z*-score of the largest connected component size of the respective drug targets relative to random expectation. (**E**) Comparison of the interactome overlap between two drug modules and the side effects they are associated with. Drugs that have at least one common side effect are characterized by more overlapping interactome modules compared to drugs that do not share any side effects. (**F**) Interactome overlap versus number of shared side effects across all drug pairs. Bars in A-F indicate the mean over all measurements, error bars show the 95% confidence interval.

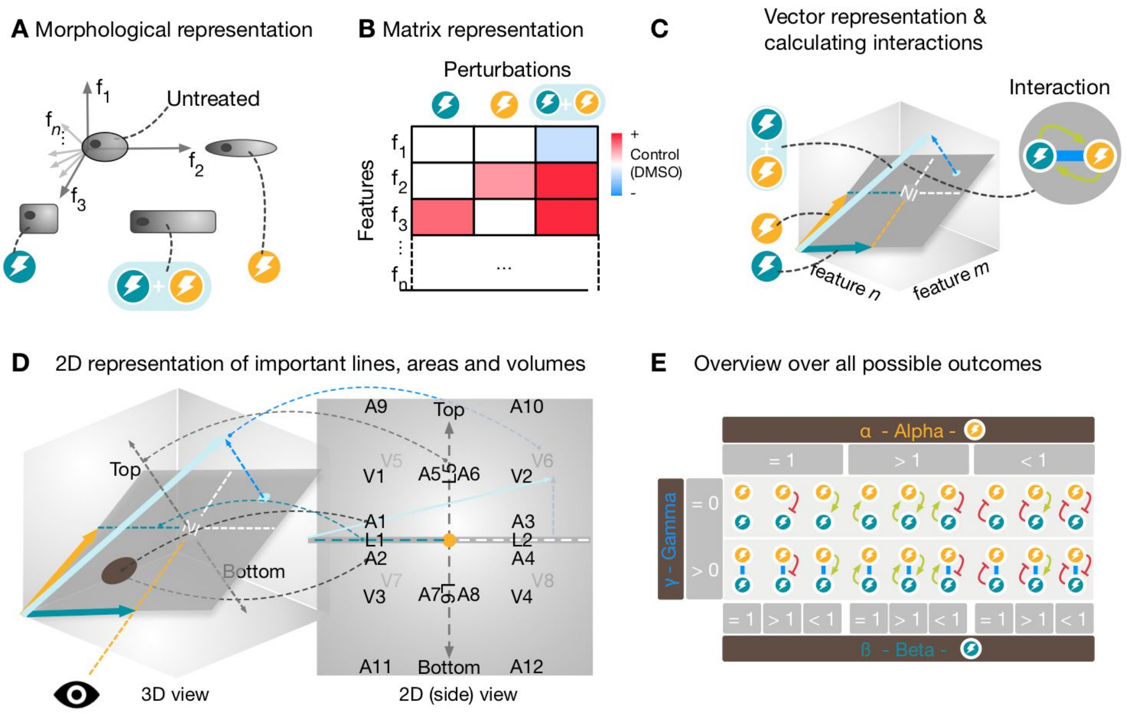

Supplementary Figure 6: Deriving perturbation interactions from cell morphology changes.

(**A**) Cell morphology readouts form the basis of our framework. Two individual perturbations (blue, orange) and their combination (light-blue) induce different morphological changes. (**B**) The cellular morphologies are transformed into a feature matrix, were rows correspond to measured features (e.g. size, eccentricity) and columns correspond to the individual and combined perturbations. All features are normalized with respect to untreated DMSO control, resulting in values smaller/larger than 0, if a feature is less/more pronounced compared to what was observed in DMSO wells. (**C**) The two single perturbations (blue and yellow) are used to span a two-dimensional plane (grey) in an *n*-dimensional feature space. The position of the combination vector relative to this plane determines the interaction type. (**D**) The top view onto the 2D plane shows 4 of the 6 1D lines (L), 12 2D areas (A) and 4 of the 8 3D volumes (V). Not visible are two lines along the field of view (yellow dashed line) and the white line that follows afterwards. From this point of view the the combination vector lies within V6 which lies behind V2. (**E**) Overview of all possible α,β and γ values and the corresponding interaction patterns.

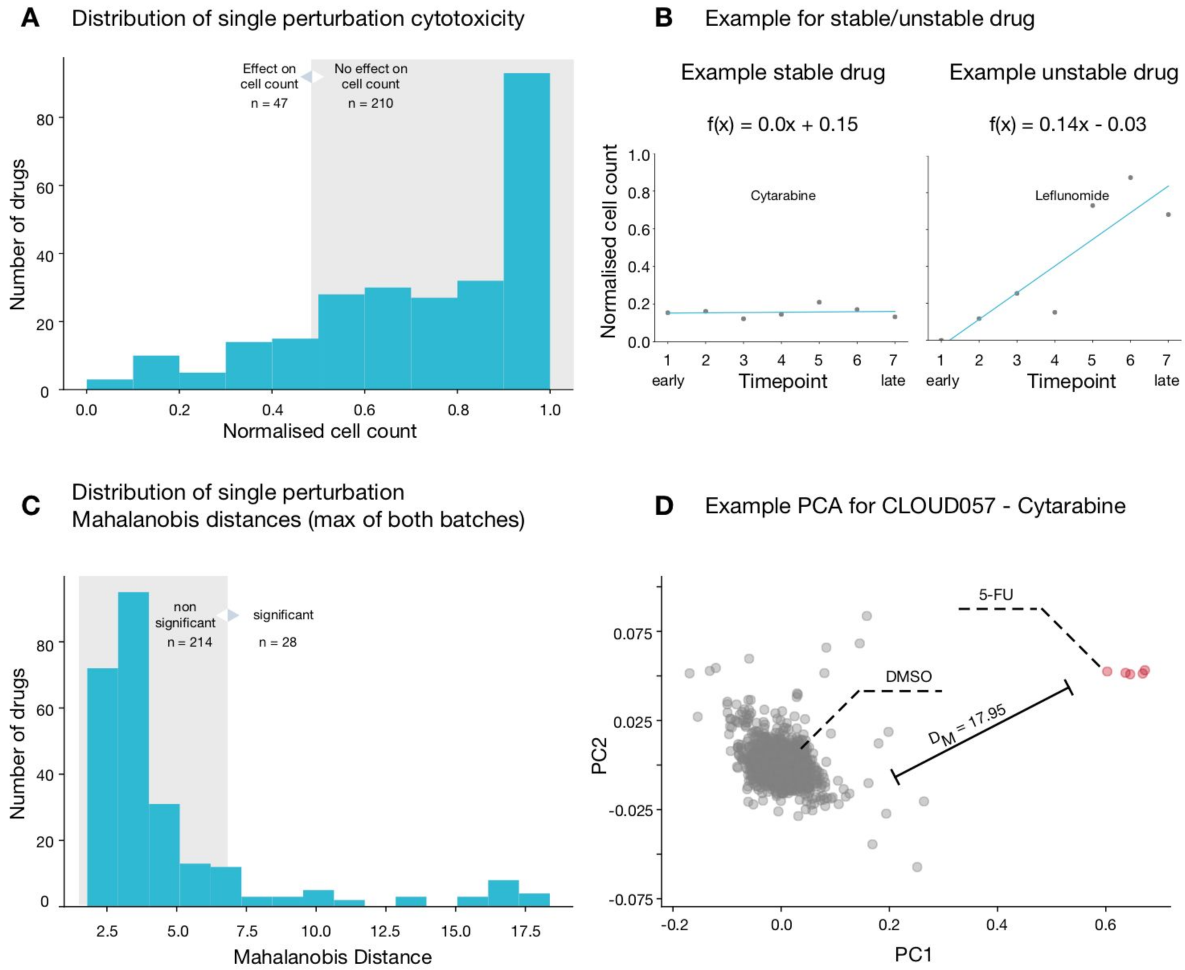

Supplementary Figure 7: Identifying significant perturbations.

(**A**) Distribution of normalized cell counts (0 = no cells, 1 = confluent). (**B**) Example regressions for a stable (cytarabine) and an unstable drug (leflunomide). Unstable drugs were excluded from the consecutive analysis. (**C**) Distribution of single perturbation Mahalanobis distances D_P. Drugs with an $D_P > 7$ are considered as significant perturbations. (**D**) Example PCA representation for 5-FU (red) compared to all DMSO wells (grey).

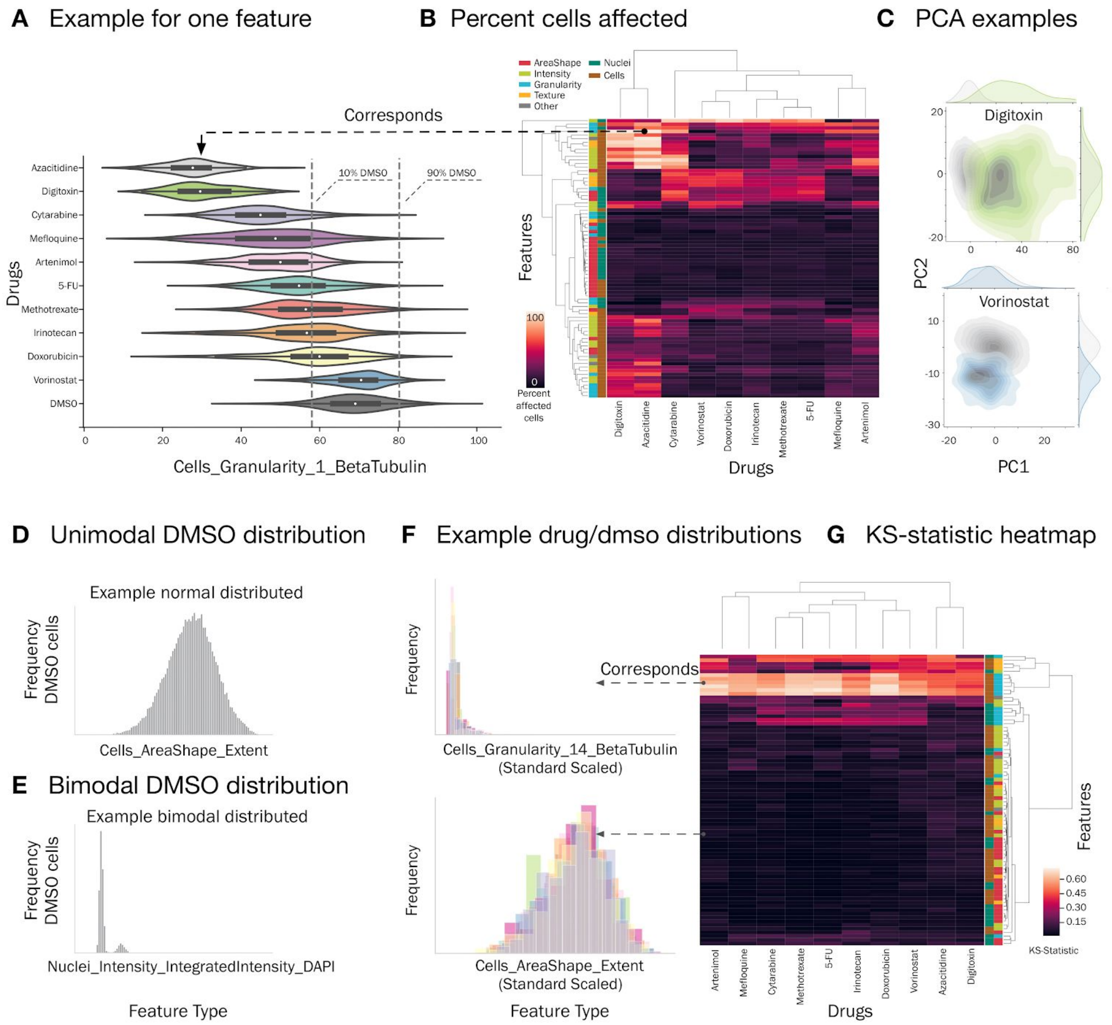

Supplementary Figure 8: The heterogeneity of cellular response to perturbations.

(A) Distribution of the values of a specific morphological feature across all cells treated with the same drug. The ten drugs are shown that induced the strongest morphological changes. **(B)** Heatmap showing for each feature/drug pair the percentage of cells that are characterized by feature values outside the 10/90 percentile range of the respective DMSO treated cells. Feature location (i.e., cell or nucleus) and feature types cluster together. **(C)** Principal Component Analysis (PCA) plot showing the morphological heterogeneity of cells treated with two exemplary drugs and their overlap with DMSO treated cells (compare with Fig. 3C in the main text). All analysed drugs overlap with DMSO to a certain degree. **(D,E)** Two example feature distributions for DMSO treated cells. The majority of features are characterized by unimodal, bell-shaped distributions (D), except for a few nuclei features, which exhibit bimodal distributions(E). **(F)** Comparison of the centered and rescaled feature value distributions of drug treated and DMSO treated cells for two exemplary features. **(G)** Comparison between the shapes of the feature value distributions of drug and DMSO treated cells using the Kolmogorov-Smirnov test statistic. For the majority of features no striking differences can be found, indicating that the emergence of bimodality is rare.

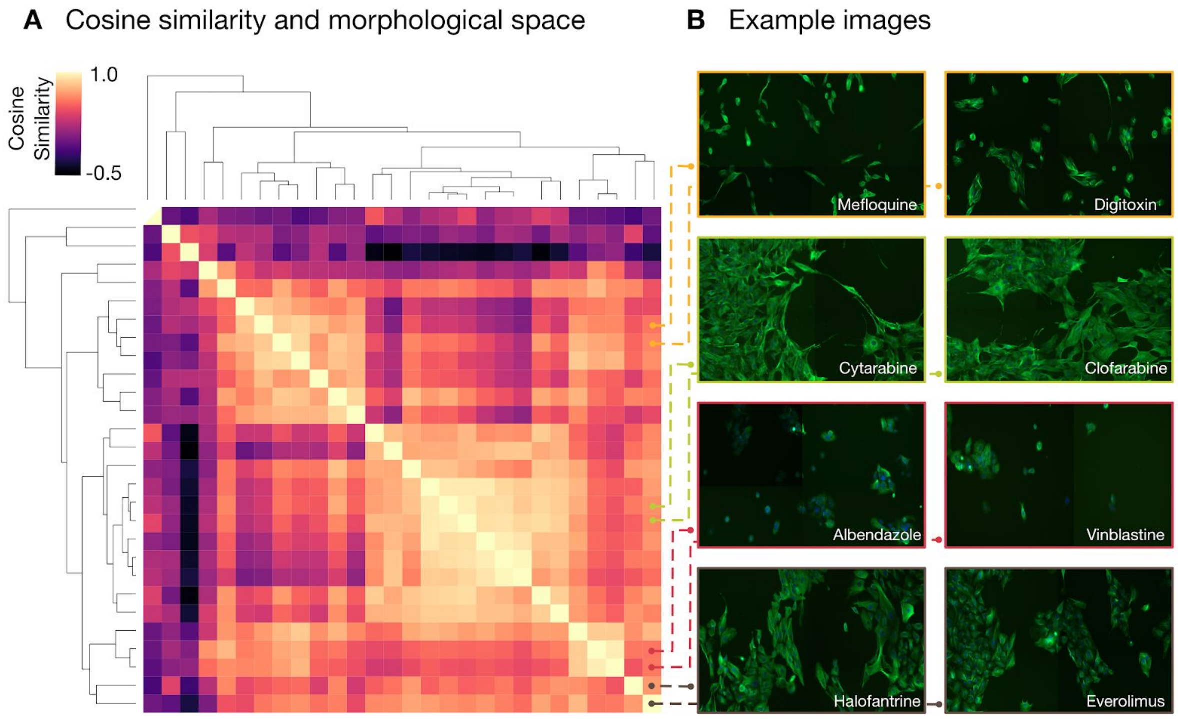

Supplementary Figure 9: Cosine distance and visual similarity.

(A) Heatmap of pairwise cosine similarities between the cell morphologies induced by the 28 drugs with the strongest effects. Drugs with similar MOA also tend to cluster within the heatmap. **(B)** Example images showing the cell morphologies of four drug pairs with high cosine similarity. High cosine similarity tends to correspond to visual similarity.

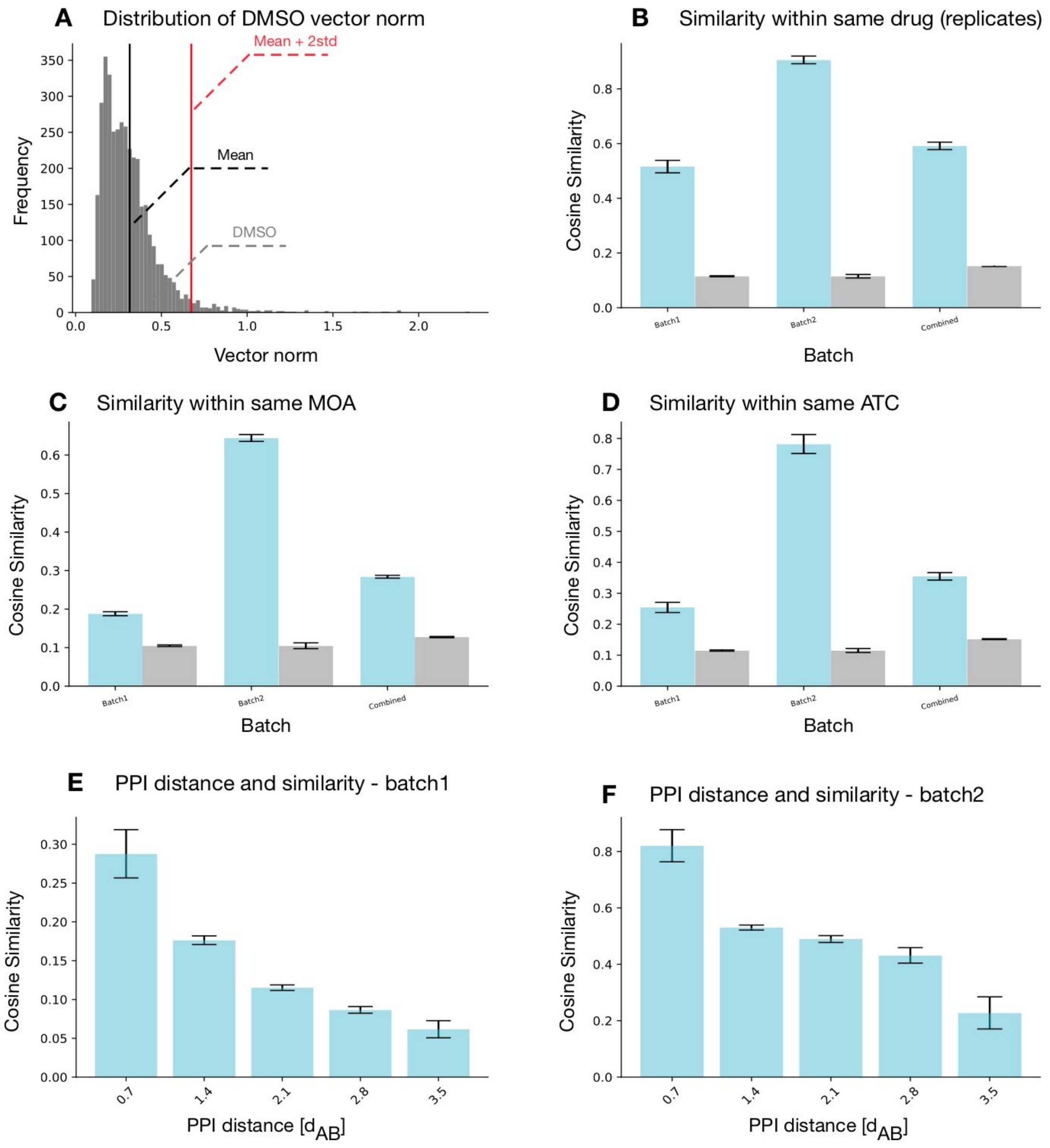

Supplementary Figure 10: Connecting morphological and biological similarity.

(**A**) Calculation of the significance cutoff for individual wells that was used to analyse morphological and biological similarity. We used the mean vector length plus two standard deviations (red line) of DMSO treated wells (grey). (**B**) Similarity between two wells treated with the same drug (blue) and two randomly picked drugs (grey). (**C**) Similarity between two drugs annotated with the same mechanism of action (blue) compared to two randomly picked drugs (grey). (**D**) Similarity between two drugs with same ATC indications compared to two randomly picked drugs. (**E**, **F**) Similarity between two drugs binned by their interactome-based distance d_{AB}. Bars in B-F indicate the mean over all measurements, error bars show the 95% confidence interval.

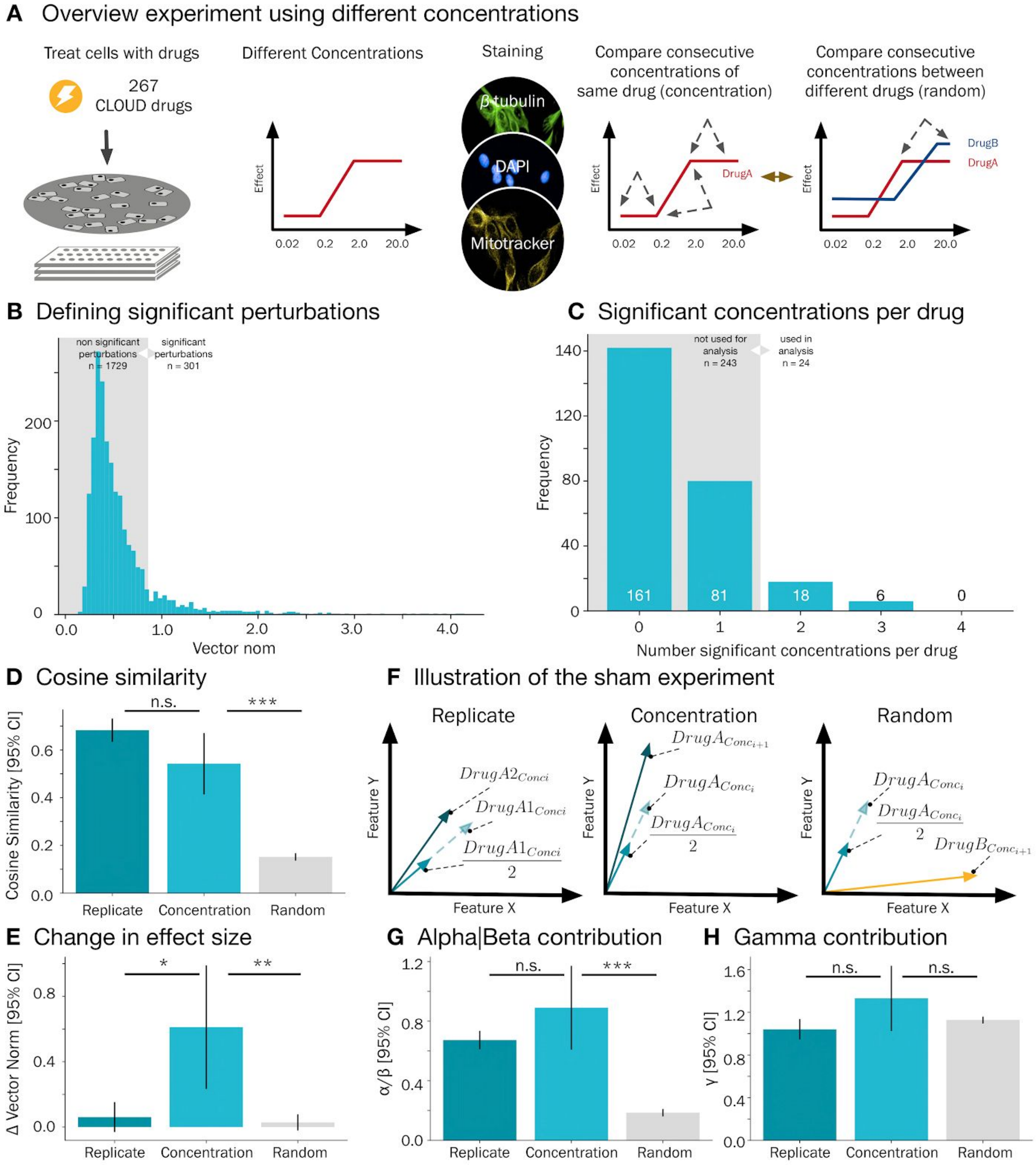

Supplementary Figure 11: Morphological profiles across different drug concentrations.

(**A**) Overview of the drug concentration imaging screen. Cells were treated with 267 CLOUD drugs in four concentrations (0.02, 0.2, 2.0 and 20µM) and two replicates using the experimental HTS/HCS pipeline introduced in SM 3. Results from consecutive concentrations of the same drug were then analysed and compared to results from randomized drugs. **(B)** Distribution of the norms of the morphological feature vectors across all treatments. Significant perturbations were defined by using the mean + 0.5 standard

deviations of all vector norms as threshold. **(C)** Histogram showing the number of concentrations in which a particular drug showed a significant effect. **(D,E)** Comparison between a pair of replicates of a drugs at a given concentration (‘replicates’), pairs consecutive concentrations of the same drug (‘concentration’) and random drug pairs (‘random’): (D) Distribution of pairwise cosine similarity as a measure of collinearity of the respective feature vectors; (E) Difference in feature vector length (i.e. vector norm), ΔVN, as a measure of change in effect size. **(F)** Illustration of the computational “sham experiment” for applying the high-dimensional interaction framework to calculate α, β and γ values for drugs at different concentrations. **(G,H)** Comparison of the distributions of α, β (G) and γ (H) values that were extracted from the sham experiment for the same drug pairs as in (D,E). Bars in D-E & G-H indicate the mean over all measurements, error bars show the 95% confidence interval. (*, ** and *** denotes *P*-values < 0.05, 0.01 and 0.001, Mann-Whitney U test).

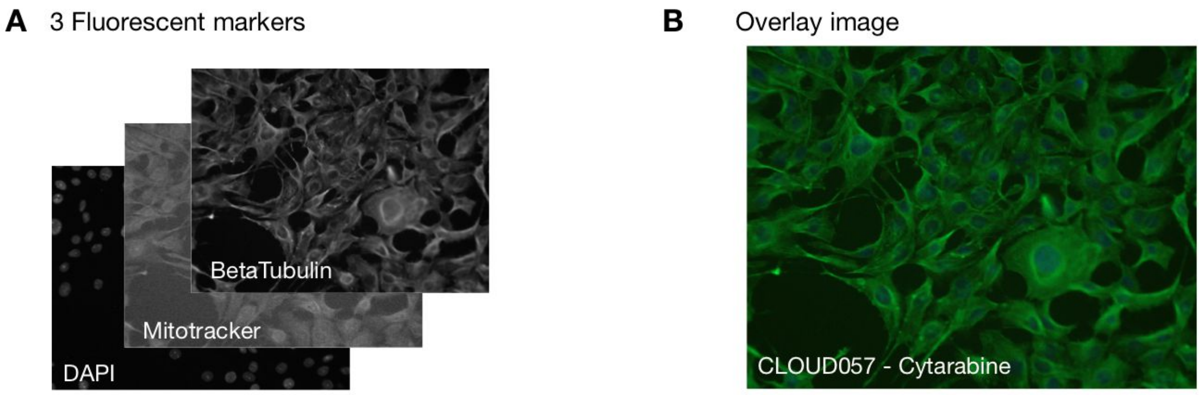

Supplementary Figure 12: Example images for MCF-10A cells treated with cytarabine.

(**A**) The three individual channels as grey scale images that have been used for feature extraction: (*i*) DAPI (blue), (*ii*) Mitotracker (red) and (*iii*) BetaTubulin (green). (**B**) Colorized overlay of the three channels combined.

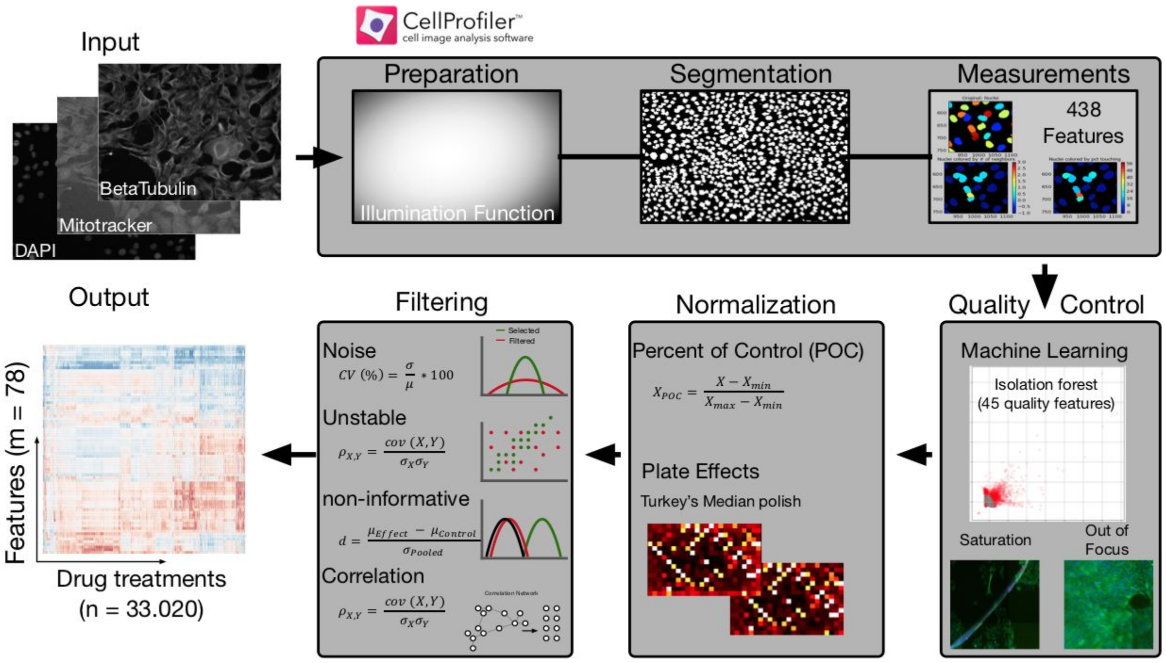

Supplementary Figure 13: Overview of the image analysis and feature extraction pipeline.

The three individual images are first corrected for uneven illumination, then segmented and features are extracted. Outlier images are removed using an isolation random forest method, the remaining image features are normalized. After filtering for robust, informative and non-redundant features, all 33,020 drug perturbations are represented in a 78-dimensional feature space.

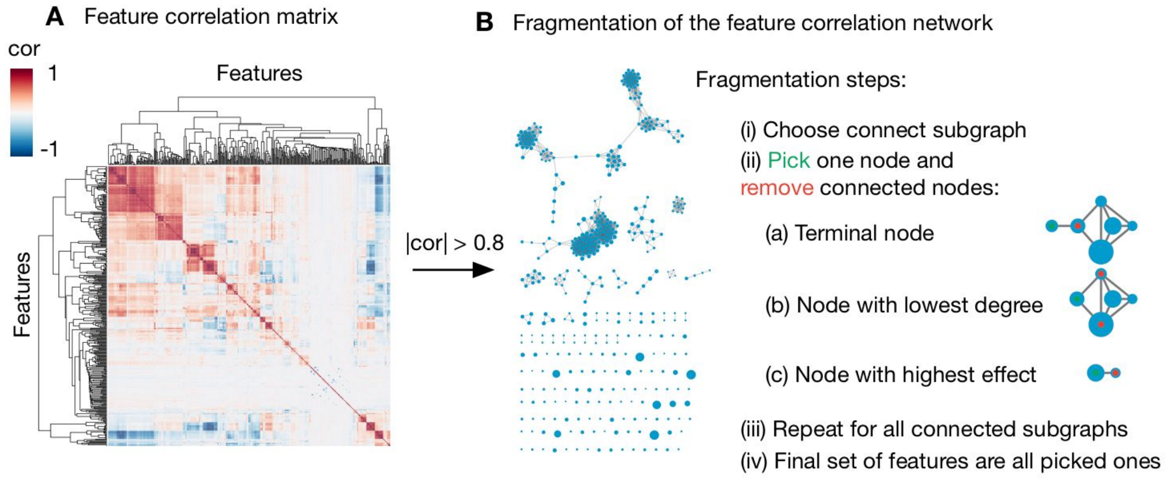

Supplementary Figure 14: Network-based approach for removing correlating features.

(**A**) Many of the 438 extracted cellular features show a strong correlation with other features, visible as clusters in the pairwise feature correlation matrix (using Pearson's ρ). (**B**) We first create a correlation network, where nodes correspond to features and edges to a correlation |ρ|>0.8. Node size corresponds to the effect size of the corresponding feature. To remove all correlation between the features while at the same time keeping the maximum amount of features, we applied the following network fragmentation algorithm: (*i*) Select one of the connected subgraphs. (*ii*) Select nodes and remove all its neighbors according to the following procedure: (a) Select all terminal nodes, i.e., nodes with only one neighbor (degree *k*=1). (b) If no terminal nodes are available, select node with the lowest degree *k*. If there are several nodes with the same *k,* perform step (a) on the given subgraph. (c) If all nodes have the same degree, choose the node with the largest effect size. (*iii*) Repeat until all nodes are either selected or removed. (*iv*) The set of all selected nodes and nodes with degree *k*=0 represents the final set of non-correlating features.

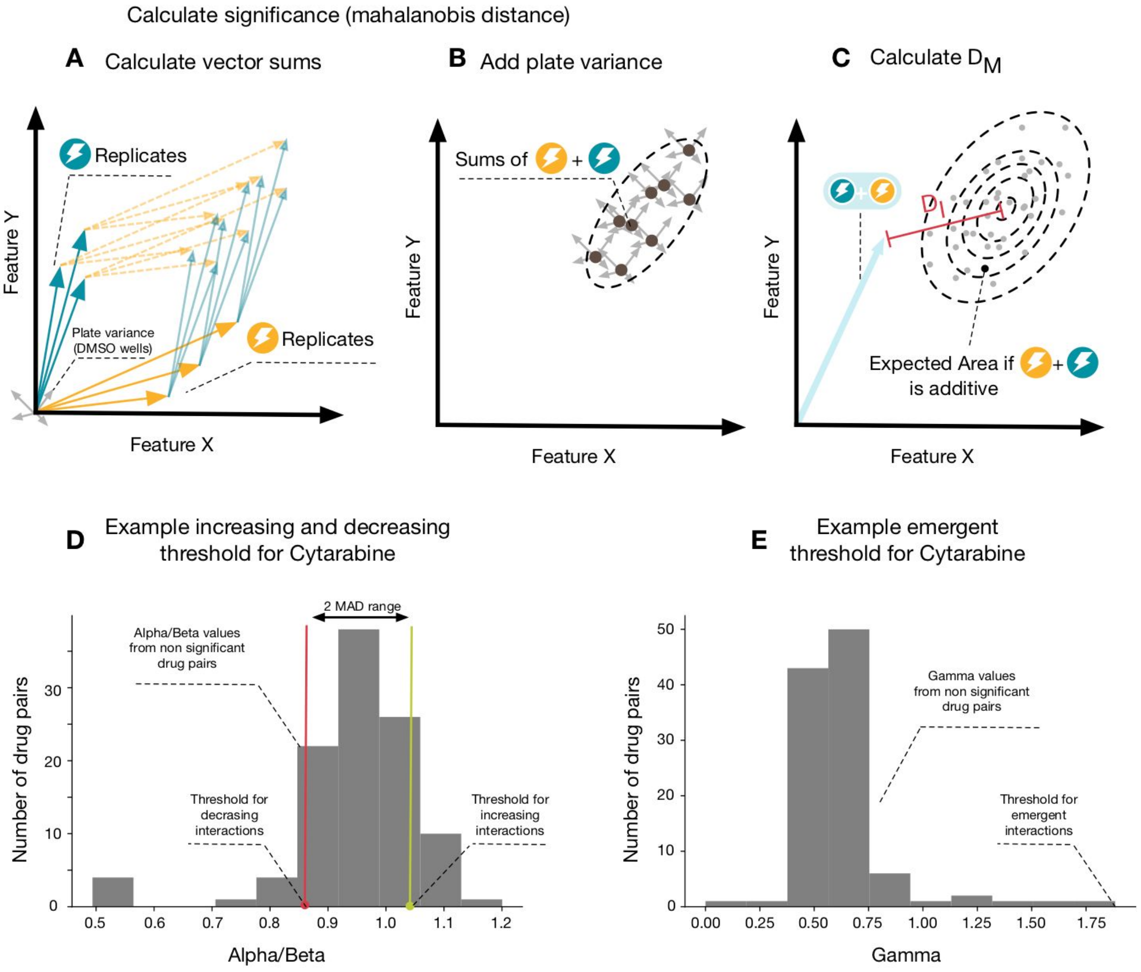

Supplementary Figure 15: Calculating the interaction thresholds.

(**A**) All possible combinations of vector sums across the replicates of the two single perturbations are calculated, resulting in ca. 30 points. (**B**) To account for random variability among the wells and to ensure that only strong and robust interactions are identified, we further superimpose the variability observed among the ~30 DMSO wells on top of the previously calculated points, resulting in ca. 900 points that define the density area of non-interaction in the *n*-dimensional feature space. (**C**) The Mahalanobis distance D_I is used to quantify the distance between the observed combination point (blue arrow) and the area on non-interaction. (**D**) Drug pairs with $D_I < 3$ are defined as non-significant and subsequently used to calculate drug specific effect size thresholds for positive/negative interactions. Only values of α and β that are more than 2 mean absolute deviations away from the median among the non-significant pairs are considered as actual interactions. (**E**) As the undirected emergent interactions are of a fundamentally different nature than the directed positive/negative interactions, we calculate their respective thresholds separately. For emergent interaction, we use the largest γ among all non-significant drug pairs of a specific drug as threshold.

Supplementary Methods

CLOUD library characterization

Exhaustive screens of drug pairs are fundamentally limited by the combinatorial explosion of possible combinations: Only the 9,700 active ingredients that have been approved world-wide, for example, would lead to more than 45 million combinations, while the theoretical drug-like chemical space is estimated to contain up to ~10^{60} structures [1–3]. Here, we built on the previously established CeMM library of unique drugs (CLOUD) that was designed to capture as much as possible of the chemical and biological diversity of all US Food and Drug Administration (FDA) approved drugs [4]. The library consists of 314 drugs that are easily delivered in a single 384-well plate in concentrations related to their therapeutic plasma levels to make high-throughput screening as efficient as possible. The library was derived using data mining techniques to cluster FDA approved drugs into classes of structural and activity similarity (Supplementary Fig. 2A). Choosing representative compounds by taking also ease of availability into account resulted in 244 small molecules. The CLOUD further contains 35 drugs whose biological target is still unknown and 35 molecules representing the active forms of prodrugs, i.e., compounds that become pharmacologically active after being administered and metabolized. For our analysis and subsequent screen we used a slightly smaller version of the CLOUD consisting of 267 drugs based on their availability at the time of usage. The concentration of each drug was picked individually based on its reported human peak plasma concentrations to encourage High Throughput Screen (HTS) at pharmacologically relevant doses (Supplementary Fig. 2B). (see Supplementary Data 2).

Investigating cell population heterogeneity

Individual cells may respond differently and to varying degrees to a drug induced perturbation. As a consequence, population wide averages of a given morphological feature reflect both the heterogeneity in terms of the phenotypic effect of a drug and in terms of the affected proportion of cells. To quantify the relative contribution of these two effects, we examined the top ten drugs with the largest morphological impact more closely. Supplementary Figure 15A shows the distributions of the values of a particular feature across cells treated with the ten drugs, as well across DMSO treated cells. The distributions indicate a broad variability of responses, both between different treatments, but also among

cells treated with the same drug. The extent of variability among drug treated cells is similar to the variability among control cells. All drugs show at least some overlap with the DMSO control. We quantified this overlap for each of the 78 features that qualified our requirements (see Methods section in the main text) and each drug by calculating the percentage of drug treated cells that exhibited a feature value outside the 10/90 percentile range of the respective feature observed among the DMSO treated cells. The results summarized in Supplementary Fig. 15 B show that each drug affect specific features and to varying degrees. While there are few feature/drug pairs, for which the feature values across drug treated cells are almost completely separated from the values across DMSO treated cells (corresponding to bright areas with values close to 100% in Supplementary Fig. 15B), each of the respective drugs also exhibits features that show little to no difference compared to the control cells. This is also reflected in the PCA analysis of all features for two exemplary drugs shown in Supplementary Fig. 15C. The green and blue areas represent the morphological heterogeneity of cells treated with two drugs, the grey area the heterogeneity of DMSO treated cells.

We next investigated whether some of this observed heterogeneity can be attributed to the existence of subpopulations within the same well that exhibit clearly distinct morphologies. This could indicate, for example, that only a fraction of all cells responded to a particular treatment. We first inspected the distribution of feature values across DMSO treated cells, finding that the vast majority of features are characterized by unimodal, bell-shaped distributions, thus reflecting a continuum of values without clearly distinct subpopulations (Supplementary Fig. 15D). The only exceptions were nuclei intensity features, whose distributions exhibited a mild bimodality (Supplementary Fig. 15E). The position of the two maxima suggests that they represent cells in different phases of the cell cycle, i.e. containing 2C and 4C DNA amount, respectively. We next tested whether any of the drug treated populations showed feature distributions with different shapes compared to the respective distributions of the DMSO controls. To this end, we centered and rescaled all distributions by removing the mean and scaling to unit variance before computing the Kolomogorov-Smirnov test between the respective DMSO and drug treatment distributions (Supplementary Fig. 11F). The results summarized in Supplementary Fig. 15G show that for the vast majority of drug/feature pairs there are no significant differences between the shape of the DMSO and the shape of the drug treatment distributions. Only a few granularity features showed larger differences, which correspond to an increase in the number of cells with a zero value. This increase is likely the result of the size of the virtual probe that is used to calculate the respective features and does not reflect the emergence of a separate cell subpopulation.

Taken together, we found no evidence for the widespread existence of distinct cell populations. We conclude that the observed morphological heterogeneity among cells of the same treatment is largely driven by effects at the cellular level, instead of reflecting an incomplete penetrance of the perturbation at the cell population level, i.e. a drug affecting only a fraction of all cells.

Comparing feature vector similarity and visual similarity

Figure 3C of the main text shows that closeness of two drugs in the two dimensional PCA projection of the 78-dimensional morphological space does not always correspond to visual similarity of the respective cellular phenotypes. To test whether the identified morphological features reflect actual visual similarity between two images we calculated the cosine distance between the feature vectors of all significant single drug treatments. Supplementary Figure 8 shows the resulting heatmap and four drug pairs that lie next to each other when performing clustering based on their cosine similarity (see main methods section). We found that cosine similarity and visual similarity generally agree well with each other. We further noticed that drugs with the same MOA tend to cluster together, similar to the results presented in Fig. 3C-F in the main text. For example, Clofarabine and Cytarabine are both antimetabolites, whereas Albendazole and Vinblastine are both tubulin inhibitors.

Overview databases

In addition to the target data described in section in the main method section, we compiled diverse annotation data for the 242 drugs in the perturbome using nine public databases, see Supplementary Data 11 for a full list.

DrugBank

DrugBank is an encyclopedic web repository aiming to provide complete biochemical and pharmacological data about drugs, including biological mechanisms and targets information [5]. Most of the contained information is curated from the research literature. Currently, DrugBank lists 10,376 drug entries and 577,712 interactions between them. We used version 5.0 of DrugBank [https://www.drugbank.ca, downloaded April, 2018], parsed the drug interaction information from the provided XML file and compiled an edgelist of drug identifier combinations with additional information about their respective (*i*) targets (i.e., proteins to which a given drug binds, resulting in an alteration of the normal function of the bound molecule and a desirable therapeutic effect), (*ii*) enzymes (i.e. proteins which catalyze

chemical reactions involving the given drug), (*iii*) transporters (i.e. membrane bound proteins which shuttle ions, small molecules or macromolecules across membranes, into cells or out of cells), (*iv*), carriers (i.e. secreted proteins which bind to drugs, carrying them to cell transporters, where they are moved into the cell.), (*v*) ATC (Anatomical Therapeutic Chemical Classification System) indications [6] (i.e. organ or system on which the drug acts and the therapeutic property), (*vi*) drug interactions (with which other drugs are medical adverse drug reactions known). The number of drug interactions involving an effective overdose of one of the drugs was calculated by parsing the interaction identifier strings for the keywords 'increase' and 'increased' or 'decrease and 'decreased', respectively. Out of the 396.451 interactions, 276.527 (70%) show an increase in effect compared to 119.591 (30%) interactions that are classified as decreasing.

MSigDB

The molecular signature database (MSigDB) [7,8] offers a collection of canonical pathways and experimental signatures [http://software.broadinstitute.org/gsea/msigdb, version 6.2]. Overall, It contains 17,810 gene sets that are divided into 8 major collections. We used the collections C2 (curated gene set; chemical and genetic perturbations) and C5 (gene ontology gene sets).

Gene Ontology (GO)

The gene ontology [http://geneontology.org/] offers a controlled vocabulary of gene and gene product attributes [9]. The three branches of gene ontology cover molecular function (MF), cellular component (CC) and biological process (BP). The gene sets we used were obtained from the MSigDB, see above.

HIPPIE

The Human Integrated Protein Protein Interaction Reference (HIPPIE) [10] is a database for functionally annotated and confidence scored human protein-protein interactions collected from various source databases and studies (that have not been fully covered by the other databases yet). The source databases include IntAct, MINT [11] and BioGRID [12]. We used version 2.1 [http://cbdm-01.zdv.uni-mainz.de/~mschaefer/hippie/index.php, downloaded on Jul 18, 2017], which contains 324,777 interactions. Filtering for proteins and interactions with at least one literature reference results in an interactome network containing 16,393 proteins and 309,365 interactions (see Supplementary Fig. 1).

KEGG

The Kyoto Encyclopedia of Genes and Genomes (KEGG) is a resource for understanding high-level functions and utilities of biological systems, such as the cell, the organism and the ecosystem, from molecular-level information, especially large-scale molecular datasets generated by genome sequencing and other high-throughput experimental technologies [13]. We included all 330 KeGG pathway gene sets in our analysis [https://www.genome.jp/kegg/pathway.html, accessed November 2018].

DisGeNET

DisGeNET [http://www.disgenet.org/, accessed November 2018] is a discovery platform containing one of the largest publicly available collections of genes and variants associated with human diseases [14]. DisGeNET integrates data from expert curated repositories, GWAS catalogues, animal models and the scientific literature. We only included the following sources: HPO, CTD, PSYGENET, ORPHANET and UNIPROT, resulting in ca. 130,821 gene-disease associations.

Disease Ontology

Disease Ontology [http://disease-ontology.org/, downloaded April 2018] is a standardized ontology for human disease with the purpose of providing the biomedical community with consistent, reusable and sustainable descriptions of human disease terms, phenotype characteristics and related medical vocabulary disease concepts [15]. Parsing the ca. 130,821 gene-disease associations using the disease ontology allowed us to link 7795 genes to 3630 diseases.

SIDER

The Side Effect Resource (SIDER) [http://sideeffects.embl.de/, downloaded November 2018] contains information on marketed medicines and their recorded adverse drug reactions [16]. The information is extracted from public documents and package inserts. The available information include side effect frequency, drug and side effect classifications, as well as links to further information, for example drug–target relations. We downloaded the database Nov. 2018 and used PubChem identifier to map our CLOUD drugs to the database.

Offsides

The Offsides database is a resource for off-label effects that are not not listed by the FDA [17]. The version used in this study was obtained from the Offsides Webpage [http://tatonettilab.org/, downloaded April, 2018] and consists of 438,801 side effects for

1332 drugs and 10,097 adverse events. We parsed the information from the downloaded text file and used again PubChem identifiers to map TwoSides identifiers (see below) to our CLOUD drugs.

TwoSides

TwoSides is a comprehensive source of polypharmacy adverse drug reactions (ADRs) for drug combinations [17]. The version used in this study was obtained from the TwoSides Webpage [http://tatonettilab.org/, downloaded April 15, 2018] and contained of 63,473 ADRs between 645 drugs. Interactions in the TwoSides database are restricted to those that cannot be unambiguously ascribed to either drug alone. We performed the same parsing as for the Offsides and SIDER database.

Morphological effects across drug concentrations

The effects of drugs generally depend on their dosage. Most commonly, sigmoidal functions are used to model dose-response curves that interpolate between no effect observed at very low dosages and a saturation of the effect observed at high dosages. To test whether the basic principles of our high-dimensional framework can also be applied across different concentrations, we performed a second imaging screen of all CLOUD drugs in four different concentrations ranging from 0.02 to 20.0 µM (see Supplementary Fig. 10A). Specifically, we aimed to test whether (i) the morphological effects of a drug at a higher concentration are more pronounced, yet qualitatively similar to the effects observed at a lower concentration and (ii) whether an increase in concentration is associated with an increase in emergent effects. Applying the same filtering and feature extraction protocol as described in the main methods section resulted in 2030 valid drug perturbations described by 49 morphological features (see Supplementary Data 14). Significant perturbations were defined by a vector norm larger than the mean + 0.5 standard deviations of all vector norms (see Supplementary Fig. 15B), representing 15% of all screened perturbations. For our subsequent analysis, we identified all drugs with significant changes in at least two concentrations, representing 9% of all drugs in the library (Supplementary Fig. 10C). To quantify the extent to which the overall morphological shape induced by a given perturbation is conserved across different concentrations, we computed the cosine similarity between the respective feature vectors. Supplementary Fig. 10D shows only a minor and statistically not significant reduction in cosine similarity among drugs of different concentrations compared to replicates, i.e. drugs of the same concentration (mean similarities S_{cos}(repl.) = 0.68 *vs.* S_{cos}(conc.) = 0.54; both are highly significant compared to values observed among random drug pairs,

S_{cos}(rand.) = 0.16). Comparing the length of the respective vectors between replicates and between different concentrations, we found that the morphological effect typically increases with increasing dosage of a drug (average difference in vector norms ΔVN(repl.) = 0.06, ΔVN(conc.) = 0.61 and ΔVN(rand.) = 0.04, see Supplementary Fig. 10E). Taken together, these results indicate that an increase in drug concentration generally leads to a more pronounced morphological phenotype, which is characterized by a vector of increased length pointing in the same direction within the morphological space.

We next tested whether these findings can also be recapitulated through the high-dimensional interaction framework introduced. To this end, we performed a computational "sham experiment" as illustrated in Supplementary Fig. 10F: To apply the formula $\alpha \vec{a} + \beta \vec{b} + \gamma \vec{n} = \vec{c}$ introduced in the main text, we interpreted both single treatment vectors $\vec{a}$ and $\vec{b}$ as the vector of a given drug at concentration *i* scaled to half of its actual length, and $\vec{c}$ as the vector of the same drug at (higher) concentration *i* +1. If the respective vectors of the two concentrations align perfectly, the combination vector should be expressed entirely through α or β (since $\vec{a} = \vec{b}$, α and β are interchangeable in this case), while γ should be zero. Large non-zero values of γ, on the other hand, indicate that the changes induced by a higher concentration of the same drug contain a significant emergent component, i.e. morphological features that are not present at the lower concentration. Supplementary Figure 10G-H compares the results of the sham experiment when using either vectors of the same drug at the same concentration (repl.), vectors of the same drug at higher concentration (conc.) or a randomly chosen drug at a higher concentration (rand.) as combination vector $\vec{c}$. Cells treated with the same drug at different concentrations are characterized by a moderate increase in α and β values compared to the replicates (α|β(repl.) = 0.67 *vs.* α|β(conc.) = 0.89), in both cases the values are significantly larger than those obtained from random combination vectors (α|β(rand.) = 0.21). These findings are in line with the results on the cosine similarity and vector norm differences in Supplementary Fig. 10D-E, again pointing at a general increase of the induced effect for higher drug concentrations. The values of γ were comparable in all three groups (γ(repl.) = 1.04, γ(conc.) = 1.33, γ(rand.) = 1.12, Supplementary Fig. 10H) and no significant differences were observed between different concentrations and replicates or between different concentrations and random combination vectors. This indicates that the observed γ levels reflect technical variance and biological heterogeneity, rather than representing truly emergent morphological features. As described in the main methods part, we apply conservative thresholds to account for such random variations in order to identify only robust

interactions and avoid false positives. In summary, we conclude that our general framework for identifying and classifying perturbation interactions using high-dimensional morphological readouts can also be applied across different drug concentrations, when suitable controls and conservative thresholds are used that take technical and biological variance into account.

Supplementary References

1. Huang, R. *et al.* The NCGC pharmaceutical collection: a comprehensive resource of clinically approved drugs enabling repurposing and chemical genomics. *Sci. Transl. Med.* **3**, 80ps16 (2011).
2. Bulusu, K. C. *et al.* Modelling of compound combination effects and applications to efficacy and toxicity: state-of-the-art, challenges and perspectives. *Drug Discov. Today* **21**, 225–238 (2016).
3. Sittampalam, G. S., Rudnicki, D. D., Tagle, D. A., Simeonov, A. & Austin, C. P. Mapping biologically active chemical space to accelerate drug discovery. *Nat. Rev. Drug Discov.* **18**, 83–84 (2019).
4. Licciardello, M. P. *et al.* A combinatorial screen of the CLOUD uncovers a synergy targeting the androgen receptor. *Nat. Chem. Biol.* (2017). doi:10.1038/nchembio.2382
5. Wishart, D. S. *et al.* DrugBank 5.0: a major update to the DrugBank database for 2018. *Nucleic Acids Res.* **46**, D1074–D1082 (2018).
6. Skrbo, A., Begović, B. & Skrbo, S. [Classification of drugs using the ATC system (Anatomic, Therapeutic, Chemical Classification) and the latest changes]. *Med. Arh.* **58**, 138–141 (2004).
7. Liberzon, A. *et al.* Molecular signatures database (MSigDB) 3.0. *Bioinformatics* **27**, 1739–1740 (2011).
8. Liberzon, A. *et al.* The Molecular Signatures Database (MSigDB) hallmark gene set collection. *Cell Syst* **1**, 417–425 (2015).
9. The Gene Ontology Consortium. The Gene Ontology Resource: 20 years and still GOing strong. *Nucleic Acids Res.* **47**, D330–D338 (2019).
10. Alanis-Lobato, G., Andrade-Navarro, M. A. & Schaefer, M. H. HIPPIE v2.0: enhancing meaningfulness and reliability of protein–protein interaction networks. *Nucleic Acids*

Res. **45**, D408–D414 (2017).

11. Orchard, S. *et al.* The MIntAct project--IntAct as a common curation platform for 11 molecular interaction databases. *Nucleic Acids Res.* **42**, D358–63 (2014).
12. Chatr-Aryamontri, A. *et al.* The BioGRID interaction database: 2017 update. *Nucleic Acids Res.* **45**, D369–D379 (2017).
13. Kanehisa, M., Furumichi, M., Tanabe, M., Sato, Y. & Morishima, K. KEGG: new perspectives on genomes, pathways, diseases and drugs. *Nucleic Acids Res.* **45**, D353–D361 (2017).
14. Piñero, J. *et al.* DisGeNET: a comprehensive platform integrating information on human disease-associated genes and variants. *Nucleic Acids Res.* **45**, D833–D839 (2017).
15. Schriml, L. M. *et al.* Human Disease Ontology 2018 update: classification, content and workflow expansion. *Nucleic Acids Res.* **47**, D955–D962 (2019).
16. Kuhn, M., Letunic, I., Jensen, L. J. & Bork, P. The SIDER database of drugs and side effects. *Nucleic Acids Res.* **44**, D1075–9 (2016).
17. Tatonetti, N. P., Ye, P. P., Daneshjou, R. & Altman, R. B. Data-driven prediction of drug effects and interactions. *Sci. Transl. Med.* **4**, 125ra31 (2012).
