# Supplementary material for: Mapping the perturbome network of cellular perturbations
Source: Nat Commun. 2019 Nov 13;10:5140. doi: 10.1038/s41467-019-13058-9 (PMC6853941; doi:10.1038/s41467-019-13058-9)
Supplement: Supplementary file 2 — Description of Additional Supplementary Files [file 41467_2019_13058_MOESM2_ESM.pdf]

Description of Additional Supplementary Files

File Name: Supplementary_Data_1
Description: The compiled human interactome that was used in this study. (.gml)

File Name: Supplementary_Data_2
Description: The list of the CLOUD screening library including the concentrations used. (.csv)

File Name: Supplementary_Data_3
Description: CLOUD, Drugbank, Pubchem and ChEMBL identifier for each drug. (.csv)

File Name: Supplementary_Data_4
Description: MOAs for the individual drugs. (.csv)

File Name:Supplementary_Data_5
Description: List of all annotated proteins for the individual drugs, grouped into targets, enzymes, transporters and carriers. (.csv)

File Name:Supplementary_Data_6
Description: Cell culture conditions for the chosen MCF10A cell line. (.csv)

File Name:Supplementary_Data_7
Description: The two CellProfiler pipelines used to extract morphological features. (.cpproj)

File Name:Supplementary_Data_8
Description: List of all features extracted by CellProfiler. (.csv)

File Name:Supplementary_Data_9
Description: Selected features, defining the morphological space. (.csv)

File Name:Supplementary_Data_10
Description: Perturbation Mahalanobis distances D_P for each drug and batch. (.csv)

File Name:Supplementary_Data_11
Description: Features and data sources used for the interaction predictions (.csv)

File Name:Supplementary_Data_12
Description: List of drug specific feature values. (.csv)

File Name:Supplementary_Data_13
Description: List of drug pair specific feature values. (.csv)

File Name:Supplementary_Data_14
Description: List of perturbation vectors for the concentration experiment. (.csv)

File Name: Supplementary_Data_15
Description: All diseases annotated to the targets of the individual drugs. (.csv)
