# Supplementary material for: Mapping the perturbome network of cellular perturbations
Source: Nat Commun. 2019 Nov 13;10:5140. doi: 10.1038/s41467-019-13058-9 (PMC6853941; doi:10.1038/s41467-019-13058-9)
Supplement: Supplementary file 18 — Reporting Summary [file 41467_2019_13058_MOESM18_ESM.pdf]

nature research

Corresponding author(s): Jörg Menche

Last updated by author(s): Oct 7, 2019

Reporting Summary

Nature Research wishes to improve the reproducibility of the work that we publish. This form provides structure for consistency and transparency in reporting. For further information on Nature Research policies, see Authors & Referees and the Editorial Policy Checklist.

Statistics

For all statistical analyses, confirm that the following items are present in the figure legend, table legend, main text, or Methods section.

n/a	Confirmed	
☐	☒	The exact sample size (*n*) for each experimental group/condition, given as a discrete number and unit of measurement
☐	☒	A statement on whether measurements were taken from distinct samples or whether the same sample was measured repeatedly
☐	☒	The statistical test(s) used AND whether they are one- or two-sided<br>*Only common tests should be described solely by name; describe more complex techniques in the Methods section.*
☐	☒	A description of all covariates tested
☐	☒	A description of any assumptions or corrections, such as tests of normality and adjustment for multiple comparisons
☐	☒	A full description of the statistical parameters including central tendency (e.g. means) or other basic estimates (e.g. regression coefficient) AND variation (e.g. standard deviation) or associated estimates of uncertainty (e.g. confidence intervals)
☐	☒	For null hypothesis testing, the test statistic (e.g. *F*, *t*, *r*) with confidence intervals, effect sizes, degrees of freedom and *P* value noted<br>*Give P values as exact values whenever suitable.*
☒	☐	For Bayesian analysis, information on the choice of priors and Markov chain Monte Carlo settings
☒	☐	For hierarchical and complex designs, identification of the appropriate level for tests and full reporting of outcomes
☐	☒	Estimates of effect sizes (e.g. Cohen's *d*, Pearson's *r*), indicating how they were calculated

Our web collection on statistics for biologists contains articles on many of the points above.

Software and code

Policy information about availability of computer code

Data collection	Custom code in python (v.2.7.10) was used for data collection; code is available from https://github.com/menchelab/Perturbome
Data analysis	Image analysis [CellProfiler, v3.0.0]; network visualization [Cytoscape, 3.7.1]; custom code in python (v.2.7.10) used for analysis; code is available from https://github.com/menchelab/Perturbome

For manuscripts utilizing custom algorithms or software that are central to the research but not yet described in published literature, software must be made available to editors/reviewers. We strongly encourage code deposition in a community repository (e.g. GitHub). See the Nature Research guidelines for submitting code & software for further information.

Data

Policy information about availability of data

All manuscripts must include a data availability statement. This statement should provide the following information, where applicable:

- Accession codes, unique identifiers, or web links for publicly available datasets
- A list of figures that have associated raw data
- A description of any restrictions on data availability

All data generated and analysed during this study are included in this published article (and its supplementary information files). Only exception are raw imaging files due to very large file size [larger than 3TB], which are available under https://idr.openmicroscopy.org/ accession code idr0069. More details on data availability can be found under the respective section in the main text.

Field-specific reporting

Please select the one below that is the best fit for your research. If you are not sure, read the appropriate sections before making your selection.

☒ Life sciences ☐ Behavioural & social sciences ☐ Ecological, evolutionary & environmental sciences

For a reference copy of the document with all sections, see nature.com/documents/nr-reporting-summary-flat.pdf

Life sciences study design

All studies must disclose on these points even when the disclosure is negative.

Sample size	We tested 267 individual compounds and 35,511 compound combinations. These numbers reflect (i) the practical reason to fit all compounds as well as DMSO controls on one 384 well plate, as well as (ii) the feasibility of testing all possible pairwise combinations.
Data exclusions	Images with obvious defects such as illumination, precipitation and scratches were excluded in this study, as well as images with too few cells for a robust morphology identification.
Replication	All compounds were tested in 13 replicates on different plates. The robustness of the result is described in Figs. 3E and S11B.
Randomization	The details of how random expectation was determined in the different statistical analyses are described in the corresponding sections of the supplementary material.
Blinding	No blinding was performed in this study.

Reporting for specific materials, systems and methods

We require information from authors about some types of materials, experimental systems and methods used in many studies. Here, indicate whether each material, system or method listed is relevant to your study. If you are not sure if a list item applies to your research, read the appropriate section before selecting a response.

Materials & experimental systems

n/a	Involved in the study	
☐	☒	Antibodies
☐	☒	Eukaryotic cell lines
☒	☐	Palaeontology
☒	☐	Animals and other organisms
☒	☐	Human research participants
☒	☐	Clinical data

Methods

n/a	Involved in the study	
☒	☐	ChIP-seq
☒	☐	Flow cytometry
☒	☐	MRI-based neuroimaging

Antibodies

Antibodies used	(Primary) Monoclonal Anti-β-Tubulin antibody produced in mouse [T4026] and (Secondary) Goat anti-Mouse IgG (H+L) Cross-Adsorbed Secondary Antibody, Alexa Fluor 488 [A-11001]
Validation	T4026: https://www.sigmaaldrich.com/catalog/product/sigma/t4026?lang=de&region=AT&gclid=CjwKCAjw2cTmBRAVEiwA8YMgzX3uWJjHn6zUmKBqEnBoE1OCQU5Wm924kQ0dsxMHSwykhWmG393S4hoCZv8QAvD_BwE<br><br>A-11001: https://www.thermofisher.com/antibody/product/Goat-anti-Mouse-IgG-H-L-Cross-Adsorbed-Secondary-Antibody-Polyclonal/A-11001

Eukaryotic cell lines

Policy information about cell lines

Cell line source(s)	MCF-10A [ATCC® CRL-10317™ - https://www.lgcstandards-atcc.org/products/all/CRL-10317.aspx]
Authentication	Already established cell line
Mycoplasma contamination	The MCF-10A cell line was tested negative for mycoplasma contamiation.
Commonly misidentified lines (See ICLAC register)	None
